# Three-dimensional polygonal muscle modelling and line of action estimation in living and extinct taxa

**DOI:** 10.1038/s41598-022-07074-x

**Published:** 2022-03-01

**Authors:** Oliver E. Demuth, Ashleigh L. A. Wiseman, Julia van Beesel, Heinrich Mallison, John R. Hutchinson

**Affiliations:** 1 Structure and Motion Laboratory, Department of Comparative Biomedical Sciences, The Royal Veterinary College, Hatfield, UK; 2 Department of Earth Sciences, University of Cambridge, Cambridge, UK; 3 McDonald Institute for Archaeological Research, University of Cambridge, Cambridge, UK; 4 Department of Human Evolution, Max-Planck-Institute for Evolutionary Anthropology, Leipzig, Germany; 5 Zoological Museum, University of Hamburg, Hamburg, Germany; 6 Palaeo3D, Rain am Lech, Germany

**Keywords:** Biomechanics, Palaeontology

## Abstract

Biomechanical models and simulations of musculoskeletal function rely on accurate muscle parameters, such as muscle masses and lines of action, to estimate force production potential and moment arms. These parameters are often obtained through destructive techniques (i.e., dissection) in living taxa, frequently hindering the measurement of other relevant parameters from a single individual, thus making it necessary to combine multiple specimens and/or sources. Estimating these parameters in extinct taxa is even more challenging as soft tissues are rarely preserved in fossil taxa and the skeletal remains contain relatively little information about the size or exact path of a muscle. Here we describe a new protocol that facilitates the estimation of missing muscle parameters (i.e., muscle volume and path) for extant and extinct taxa. We created three-dimensional volumetric reconstructions for the hindlimb muscles of the extant Nile crocodile and extinct stem-archosaur *Euparkeria*, and the shoulder muscles of an extant gorilla to demonstrate the broad applicability of this methodology across living and extinct animal clades. Additionally, our method can be combined with surface geometry data digitally captured during dissection, thus facilitating downstream analyses. We evaluated the estimated muscle masses against physical measurements to test their accuracy in estimating missing parameters. Our estimated muscle masses generally compare favourably with segmented iodine-stained muscles and almost all fall within or close to the range of observed muscle masses, thus indicating that our estimates are reliable and the resulting lines of action calculated sufficiently accurately. This method has potential for diverse applications in evolutionary morphology and biomechanics.

## Introduction

Over the last two decades, three-dimensional (3D) muscle reconstructions have become common practise in palaeobiology, comparative morphology and biomechanics^1–3^. Contrast-enhanced CT (e.g., “diceCT”^4^) scanning is, contrary to traditional dissection, mostly non-destructive and allows the investigation of internal structures and soft-tissues selectively and in situ^5–17^, which can provide useful data for 3D reconstructions. DiceCT facilitates measuring muscle parameters such as volume^10,12,13,18–22^ and architecture; e.g. fascicle length and orientation^18,23–28^. Accurate muscle parameters are essential for biomechanical analyses that estimate movement performances^29–33^.

In extinct taxa, volumetric muscle reconstructions have mostly focused on the jaw adductor musculature^2,3,34–42^, as their size is constrained by surrounding bones, i.e., the adductor chamber in reptiles or the zygomatic arch in mammals and, therefore, require fewer assumptions about their extent^43^; but see^33^. The appendicular musculature is usually simplified and reconstructed in form of lines of actions (LoA) for biomechanical analyses^44–54^, with only a few comparative studies conducting volumetric muscle reconstructions in the tail^55–60^. There is a clear need for a method to predict appendicular muscle parameters for extinct taxa that take their individual limb morphology accurately into account.

Volumetric reconstructions of muscles in extinct taxa are challenging, as the exact maximal 3D extents of muscle boundaries are unknown and, therefore, might be based on flawed assumptions. However, through comparison with extant taxa and osteological correlates from the outgroup-based extant phylogenetic bracket (EPB^61,62^), we can better infer the maximal dimensions of muscles, e.g., see^56,59,63–66^. Further constraints on muscle sizes can be based on the spatial organisation of epaxial, hypaxial and appendicular musculature from extant taxa^59^.

**Figure 1:**
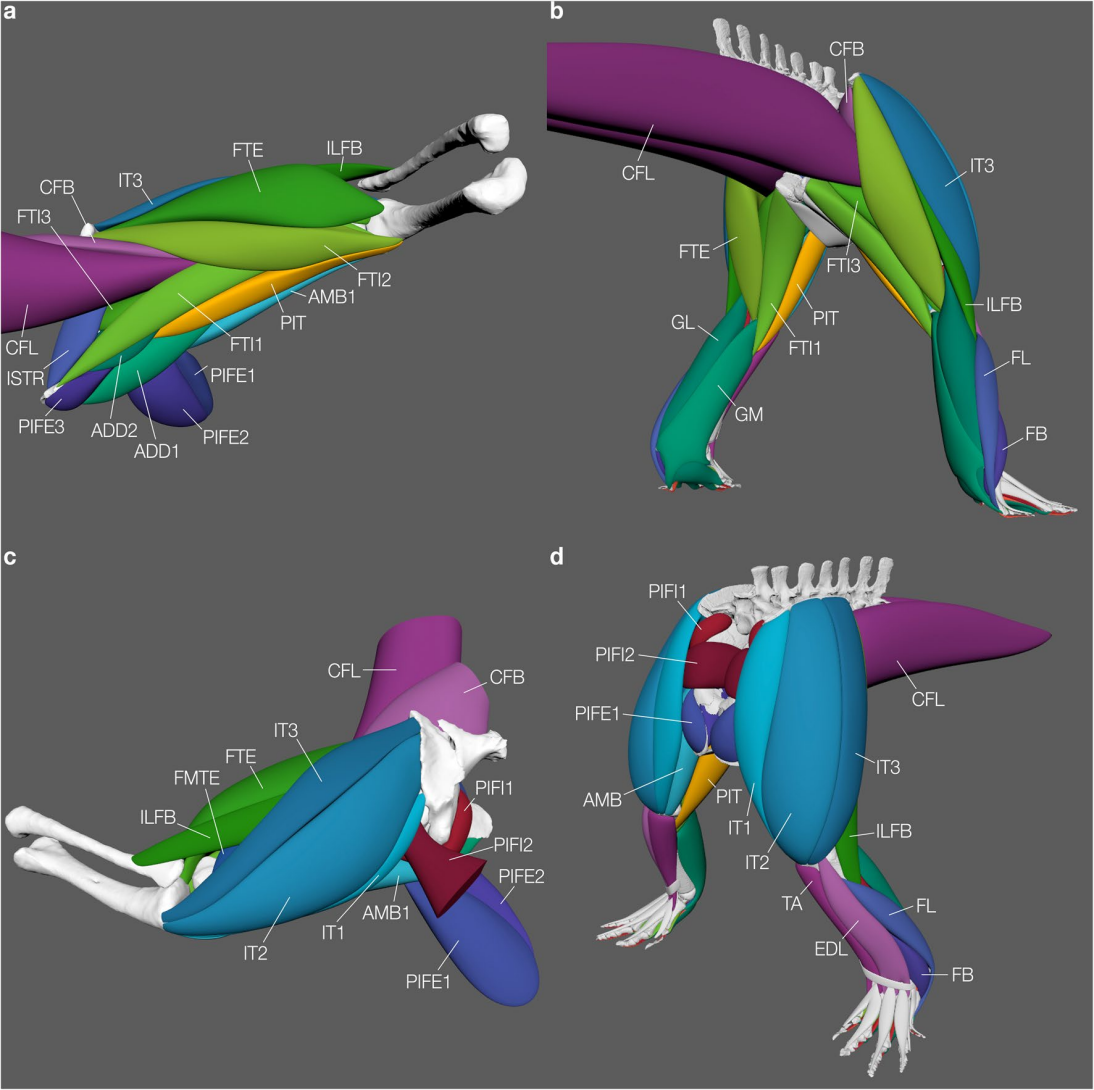
Three-dimensional polygonal muscle reconstructions. Thigh musculature of *Crocodylus niloticus* (**a,c**) and hindlimb musculature of *Euparkeria capensis* (**b,d**). A and B in oblique caudoventral view, C in oblique anterolateral view and D in craniodorsal view. Note that the CFB, CFL and PIFI2 are cut off for the Nile crocodile as the scan did not include thoracic or caudal vertebrae and could therefore not be fully reconstructed. Their insertions, however, have been modelled to prevent over-estimation of neighbouring muscles. Abbreviations: AMB(1), *M. ambiens* (1); ADD1-2, *M. adductor femoris* 1–2; CFB, *M. caudofemoralis brevis*; CFL, *M. caudofemoralis longus*; EDL, *M. extensor digitorum longus*; FB, *M. fibularis brevis*; FL, *M. fibularis longus*; FMTE, *M. femorotibialis externus*; FTE, *M. flexor tibialis externus*; FTI1-3, *M. flexor tibialis internus* 1–3; GL, *M. gastrocnemius lateralis*; GM, *M. gastrocnemius medialis*; ILFB, *M. iliofibularis*; ISTR, *M. ischiotrochantericus*; IT1-3, *M. iliotibialis* 1–3; PIFE1-3, *M. puboischiofemoralis externus* 1–3; PIFI1-2, *M. puboischiofemoralis internus* 1–3; PIT, *M. puboischiotibialis*; TA, *M. tibialis anterior*.

Here we describe and test a new method for volumetric 3D musculature reconstructions for extant and extinct taxa (Fig. 1), first applied in Díez Díaz et al*.*^59^. Instead of using non-uniform R B-splines (NURBS) to reconstruct the musculature (e.g.^33,56,58,66^), we present an approach similar to ‘box modelling’^67^. In contrast to lofting a surface over multiple rings or curves (e.g., see^33,58,60,64,68,69^) with only limited control, polygonal modelling allows adjustment of every vertex individually, to extrude new faces, as well as to create holes, which can later be filled (i.e., closed) again, allowing modelling of muscles with complex geometry (e.g., with multiple heads or tendons; see Supplementary Information). This new approach makes it possible to build the musculature in relatively low resolution and, therefore, permits quick adjustments. It further allows smoothing the muscles after completion in order to get a more detailed reconstruction with higher realism (and, presumably, precision), which can be previewed at all times during the construction process to visualize the effect of the placement of individual vertices. Importantly, we test the method’s accuracy against different data from extant taxa: both measured (via dissection) and derived from contrast-enhanced CT scanning data for crocodylian (*Crocodylus niloticus*) hindlimb muscles; and both measured and derived from surface scanning for Western lowland gorilla (*Gorilla gorilla gorilla*) shoulder muscles. We then apply our method to reconstruct the hindlimb musculature of the extinct archosauriform *Euparkeria capensis* (a distant relative of Crocodylia) to demonstrate the applicability to extinct taxa. The 3D muscle reconstructions then acts as foundation to estimate their respective LoA in an automated fashion based upon user defined input parameters in a single software package: Autodesk Maya (https://www.autodesk.com/products/maya/overview).

## Results

### Crocodile muscle mass comparison

**Figure 2:**
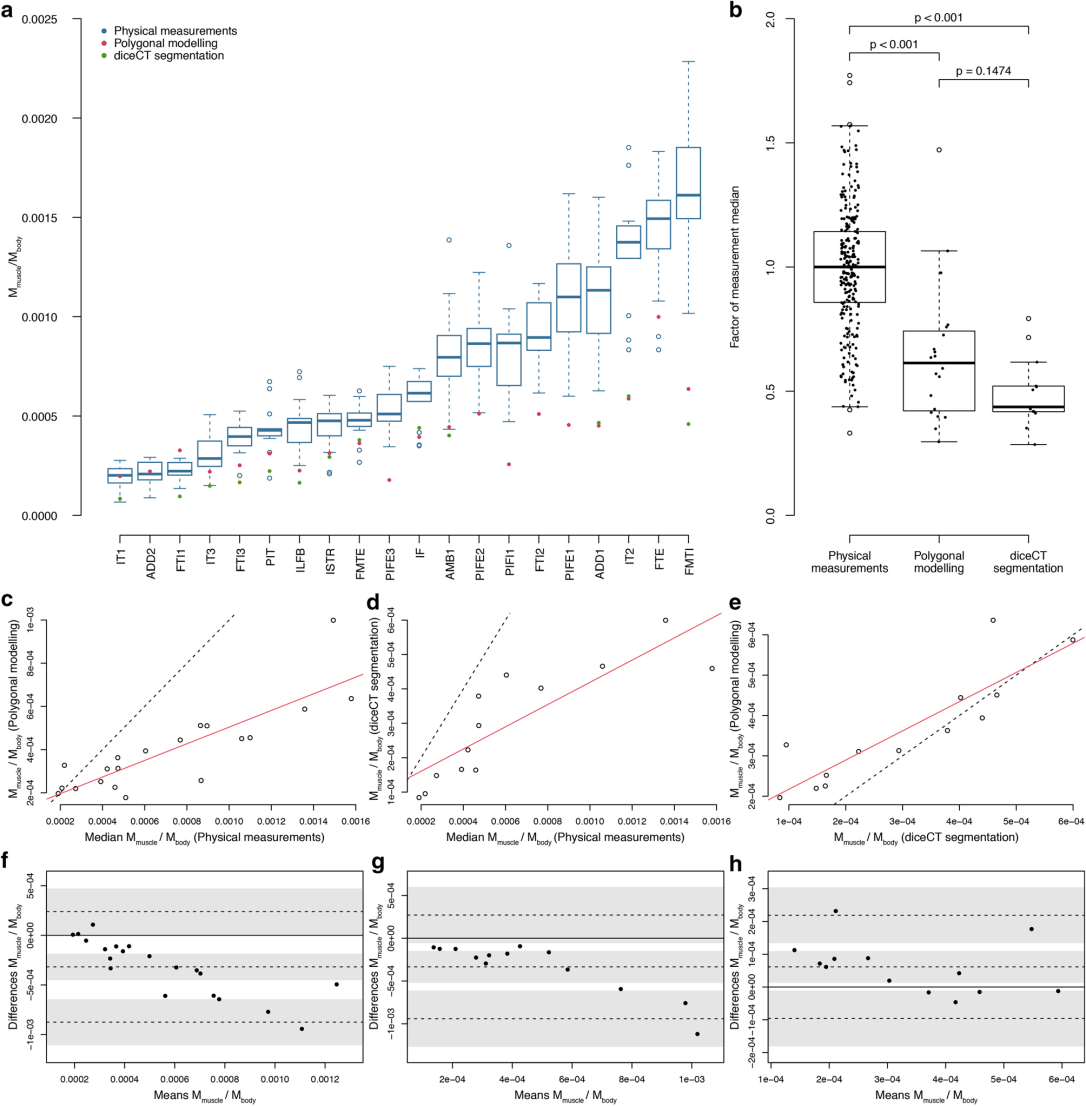
Evaluation of muscle mass estimates for the Nile crocodile. Comparison between individual muscles’ measurements and 3D reconstructions (**a**), ordered according to relative muscle mass (M_muscle_/M_body_ = muscle mass normalised by body mass). Boxplots of the physical (dissection-based) measurements in blue, muscle volume estimates from the polygonal modelling in red and calculation from diceCT segmentation in green. Note only 13 out of 20 muscles could be unequivocally segmented, hence some are missing; see Table 2 and Supplementary Information. Boxplots of the different 3D reconstructions and physical measurements (**b**). The individual muscle masses were normalised by the median of the physical measurements for each respective muscle. This shows that physical measurements of mass or volume tended to be greater than polygonal modelling and diceCT segmentation, which themselves did not significantly differ. Comparison between physical measurements and polygonal modelling (**c**) and Bland–Altman plot of the same comparison (**f**); bias -0.000318 ± 0.000133. Line of equality in (**c**) is in dashed black and linear regression model in red (y = 0.385365 x + 0. 0.000119; adjusted R^2^ = 0.6977). Comparison between physical measurements and diceCT segmentation (**d**) and Bland–Altman plot of the same comparison (**g**); bias -0.000335 ± 0.000187. Line of equality in (**d**) is in dashed black and linear regression model in red (y = 0.323222 x + 0.000096; adjusted R^2^ = 0.6967). Comparison between diceCT segmentation and polygonal modelling (**e**) and Bland–Altman plot of the same comparison (**h**); bias 0.000062 ± 0.000049). Line of equality in (**e**) is in dashed black and linear regression model in red (y = 0.723366 x + 0.000145; adjusted R^2^ = 0.7496).

As a major goal of our method is to enable the reconstruction of 3D musculature in extinct animals, we evaluated the methodology first on an animal for which we had maximally comparable data. Overall, the range of the reconstructed muscle mass estimates overlapped with the range of the physical measurements of crocodylians (Fig. 2b). However, the estimates were generally lower in mass; the polygonal modelling muscle masses were on average 38.6% lower and the diceCT-based estimates were 56.4% lower than the measured muscle masses (see Table 1; Fig. 2f,g). The Bland–Altman plots revealed under-predicted values for both the polygonal modelling (bias − 0.000318 ± 0.000133) and segmented diceCT-based muscles (bias − 0.000334 ± 0.000187) in comparison with the physical measurements. A Mann–Whitney U test showed no significant difference (W = 170, *p*-value = 0.1474) between the muscle mass estimates of the polygonal modelling approach (n = 20) and the segmented diceCT-based muscles (n = 13). However, both values were significantly lower than the body-mass-normalised physical measurements of other specimens of *C. niloticus* (n = 160, W = 4348, *p*-value < 0.001 for comparison with the former and W = 3240, *p*-value < 0.001 for the latter; Fig. 2b). Unfortunately, we could not compare the reconstructed muscle masses directly with the actual muscle masses of the same specimen, as in some previous studies, and not all muscles could be segmented reliably from the diceCT data and were thus excluded (Table 2).

**Table 1:** Comparison between measurements and muscle masses calculated from the 3D models for the Nile crocodile.

	Median	Min	Max	Within 1SD (%)	Within 2SD (%)	Within range (%)	Within range ± 20%	Number of muscles	Measurements per muscle
Measurements	1.000	0.331	1.771	70	97	100	100	20	13
Polygonal	0.614	0.296	1.472	20	60	50	70	20	1
diceCT	0.436	0.285	0.792	0	54	38	62	13	1

“Min” and “Max” = minimum and maximum relative muscle mass within dataset; *SD *standard deviation.

**Table 2:** Crocodylian thigh muscle abbreviations.

Muscle	Name	diceCT segmentation possible for *Crocodylus*
ADD1	M. adductor femoris 1	Yes
ADD2	M. adductor femoris 2	No
AMB1	M. ambiens 1	Yes
CFB	M. caudofemoralis brevis	No
CFL	M. caudofemoralis longus	No
FMTE	M. femorotibialis externus	Yes
FMTI	M. femorotibialis internus	Yes
FTE	M. flexor tibialis externus	No
FTI1	M. flexor tibialis internus 1	Yes
FTI2	M. flexor tibialis internus 2	No
FTI3	M. flexor tibialis internus 3	Yes
IF	M. iliofemoralis	Yes
ILFB	M. iliofibularis	Yes
ISTR	M. ischiotrochantericus	Yes
IT1	M. iliotibialis 1	Yes
IT2	M. iliotibialis 2	Yes
IT3	M. iliotibialis 3	Yes
PIFE 1	M. puboischiofemoralis externus 1	No
PIFE 2	M. puboischiofemoralis externus 2	No
PIFE 3	M. puboischiofemoralis externus 3	No
PIFI 1	M. puboischiofemoralis internus 1	No
PIFI 2	M. puboischiofemoralis internus 2	No
PIT	M. puboischiotibialis	Yes

Several muscles could not be segmented as the iodine staining was not homogenous and muscle boundaries could not be reliably identified for the whole muscles, thus potentially conflating their volumes with other muscles (e.g. ADD2 and PIFE3 or FTE and FTI2). These muscles were therefore excluded from the dataset. Other muscles were not fully captured in the scan window (e.g. CFB, CFL, PIFE1-3 and PIFI1-2) and could therefore not be fully segmented.

### Gorilla shoulder muscle reconstruction

**Figure 3:**
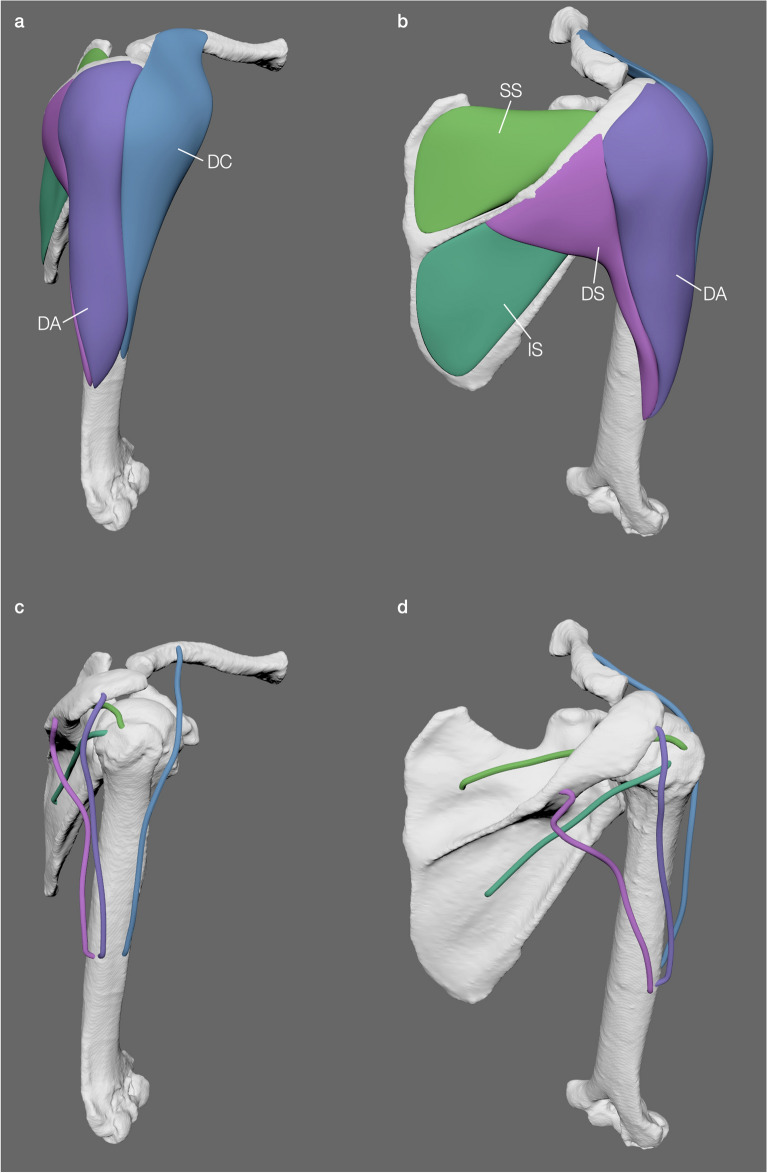
Muscle modelling and LoA estimation of the gorilla shoulder. 3D volumetric muscle models (**a**,**b**) and estimated LoAs (**c**, **d**) in lateral view (**a**,**c**) and dorsal view (**b**, **d**) respectively. Abbreviations: DA, *M. deltoideus acromialis*; DC, *M. deltoideus clavicularis*; DS, *M. deltoideus spinalis*; IS, *M. infrapspinatus*; SS, *M. supraspinatus*.

To be able to create accurate LoAs, individual 3D muscle bellies need to be reconstructed accurately and differences between dissection data and the muscle mass calculations from 3D models should therefore be minimal. We modelled five shoulder muscles of the gorilla specimen (Fig. 3). To evaluate the accuracy of the modelled muscles, their calculated masses were compared with dissection-based measurement data (Table 3) published by van Beesel et al*.*^70^. The virtual muscle masses matched the measured muscle masses with a total mass of 0.462 kg in the former and 0.475 kg in the latter, an overall underestimation of just 2.695%, indicating that the resulting LoAs (Fig. 3c,d) were also reasonable.

**Table 3:** Comparison between measurements and muscle masses calculated from the 3D models for the gorilla.

	Measured mass (kg)	Modelled volume (cm^3^)	Modelled mass (kg)	Difference (%)	LoA length (cm)
M. deltoideus clavicularis	0.063	65.405	0.069	9.576	25.434
M. deltoideus acromialis	0.166	140.047	0.148	− 10.669	21.325
M. deltoideus spinalis	0.057	51.387	0.054	− 4.001	19.733*
M. deltoideus (combined)	0.286	256.839	0.272	− 4.871	–
M. supraspinatus	0.084	79.440	0.084	0.305	15.539
M. infraspinatus	0.105	99.730	0.106	0.843	15.747
Total	0.475	436.008	0.462	− 2.695	–

The length of the muscle was measured along the LoA (arc length) of the modelled muscles and represents the length from origin to insertion and thus the total length of the muscle tendon unit (MTU).

*Note the length of the *M. deltoideus spinalis* was exaggerated due to the kink in the LoA estimation (see Fig. 3d).

### *Euparkeria* muscle reconstruction

**Figure 4:**
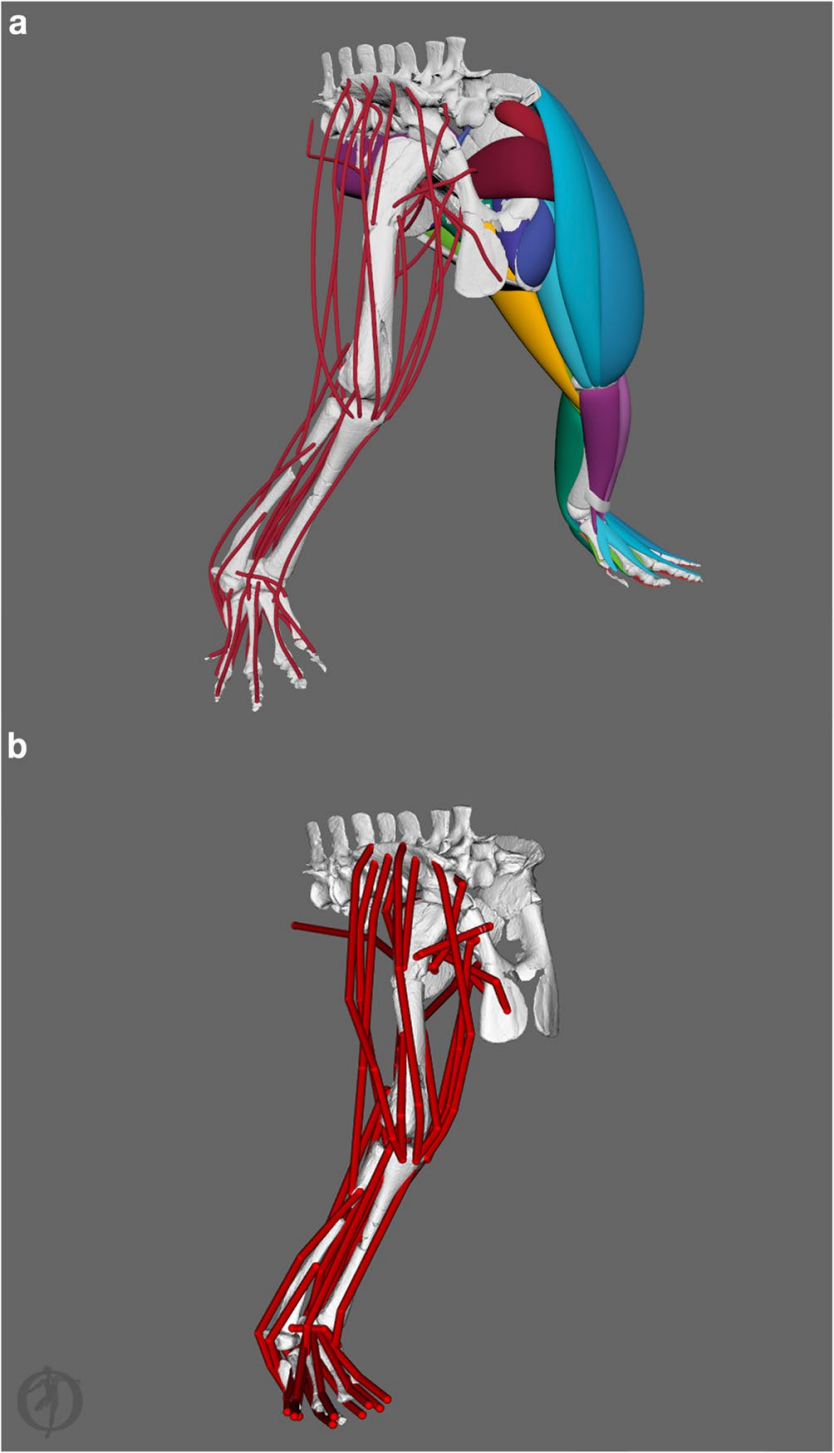
Muscle modelling and LoA estimation of *Euparkeria capensis*. (**a**) 3D volumetric muscle models and estimated LoAs. (**b**) OpenSim model with muscle paths adjusted to match the LoA based on the volumetric muscle models.

**Figure 5:**
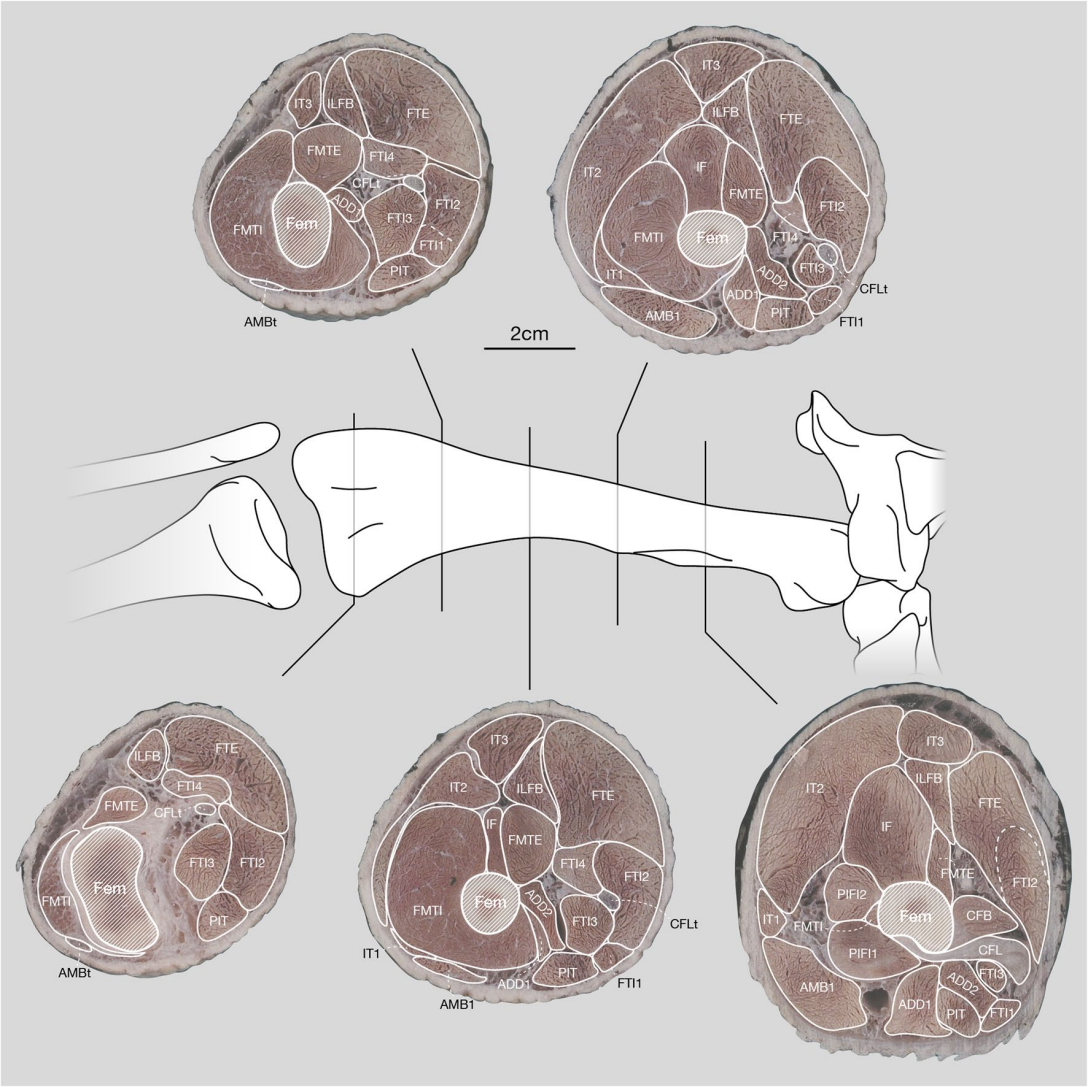
Cross-sections of a right alligator thigh with their positions indicated along the femur. For the cross-sections cranial is to the left and lateral to the top. The skeletal drawing is in cranial view and not to scale. For muscle abbreviations see Table 1; further abbreviations: AMBt, ambiens tendon; CLFt, caudofemoralis tendon; Fem, femur.

We reconstructed the complete pelvic and hindlimb musculature of a Mesozoic reptile in 3D for the first time that we are aware of (Figs. 1b,d, 4a). The reconstruction is based on muscular information from the EPB and additional *Alligator* hindlimb cross-sections (Fig. 5; Supplementary Figures S1–S3) to constrain their size and spatial organisation. Our code (provided in the Supplementary Information) successfully estimated the LoAs automatically (Fig. 4a), guided by the 3D muscle reconstructions. Calculated LoAs can then be transferred into specialist software, such as OpenSim^71^, for future biomechanical modelling and other analyses (Fig. 4b).

## Discussion

In this study, we present an *iterative polygonal modelling approach* used to virtually reconstruct 3D muscle volumes of different species. Using custom written code (See Supplementary Information), we were able to create a LoA for each individual muscle based on the respective muscle volume in a single software package with high geometric accuracy. LoAs are important for studies interested in musculoskeletal function^29,50,53,54,70,72^. For such studies, LoA reconstructions become now easily available by applying the *Maya muscle line of action estimation* code (provided in the Supplementary Information) on volumetric muscle models, either derived from diceCT scanning or reconstructed using our approach, without the need to transfer them into other software packages^73,74^. This procedure allowed us to directly establish their spatial relationships with the respective body segments, which would be valuable for subject-specific musculoskeletal models.

Our approach in combination with tomographic data allows reduction of the amount of uncertainty in the muscle reconstruction of extinct taxa (in combination with the EPB) and additionally facilitates the three-dimensional quantification of muscle geometry in extant taxa where other means are impossible; e.g. lack of contrast-stained CT/MRI-scanned specimens. It also allows estimation of muscle parameters to match best-practice workflows, e.g.^29,74–77^, and due to advantage of 3D muscle models our method can be directly combined with other methods, such as muscle discretization^78,79^, if multiple lines of action are desired, or biomechanical modelling and simulation^29,46,77,80^. Additionally, the muscle models could be directly used for finite element (FE) analyses as they are already quadrilaterally tessellated^81^, or could even act as subject-specific target meshes for fibre registration in FE models^82^. Furthermore, 3D reconstructions of the shape of individual muscles and the configuration of the musculature are enabled for extant taxa when only surface models and dissection data are available, thus facilitating further downstream analyses. Capturing surface data during a dissection, either through photogrammetry or other surface digitization techniques^1,83–87^, and reconstructing the musculature using our approach could streamline the pipeline for comparative morphological analyses^8,22,27,70,72,88^ as well as biomechanical models and simulations. Our method enables the subject-specific quantification of muscle attachment area as well as muscle shape and path estimations, information usually lost during dissection, which, however, is crucial for musculoskeletal modelling^29^.

The masses of the reconstructed *Gorilla* muscles were evaluated to ensure accuracy in the muscle belly reconstruction and thus LoA estimation for a subject-specific musculoskeletal model. Because the muscle belly reconstructions accurately describe the muscle belly shape (guided by the surface scans) and reasonably approach the muscle mass, the resulting LoA calculations for the Western lowland gorilla were, therefore, deemed accurate (Fig. 3c,d). Discrepancies in the muscle masses are relatively minor as the muscles could be guided directly with the dissection surface scans, not needing to be approximated using cross-sections. Superficial muscles of the gorilla were more difficult to accurately reconstruct than deeper muscle layers. Muscles tightly constrained by bone such as the *M. supraspinatus* and *M. infraspinatus* showed little discrepancy between modelled and measured muscle masses (Table 3). The relative position of the humerus to the scapula during surface scanning had a greater influence on the muscle shapes and resulting masses (± 10%) than for deeper muscles (± 1%). However, the overall discrepancy in the *M. deltoideus* muscle mass was only around 5% (Table 3), which might indicate that the discrepancy in the different heads can in part be explained by uncertainties where the muscle was split into subsections. For future studies we recommend fixing the bones in situ at a constant orientation for surface scanning to minimise the influence of different bone orientations on the muscle reconstructions. Nevertheless, the minor percentage differences between the modelled and measured muscle masses (Table 3) indicate the suitability and adaptability of the *iterative polygonal modelling* approach to reconstruct individual muscle bellies from surface scan data for sufficiently accurate LoA estimation.

The crocodylian muscle mass estimates of the *iterative polygonal modelling approach* overall match the masses obtained from the iodine-stained CT data (Fig. 2b), which was previously demonstrated to be an accurate method for quantifying muscle mass in avian limb muscles^21^. Potential discrepancies in the crocodile muscle mass estimates and physical measurements could be attributed to errors in the muscle modelling process; or to simplifications in the assumption of muscle composition (i.e., the 3D model assumed a homogeneous mass distribution). These differences could indicate that our crocodile specimen was either heavier than expected, thus reducing our body-mass-normalised muscle mass estimates or that simplifications in the modelling process negatively affected the muscle masses. The simplification of the muscle into quasi-cylinders results in negative spaces between the muscles that cannot be filled without intersections of the musculature elsewhere. This negative space was absent in the segmented diceCT muscles due to their higher resolution and represents a limitation of our modelling approach.

The masses of larger muscles appeared to be more difficult to estimate (Fig. 2) and were more likely to be underestimated in both the polygonal modelling (Fig. 2c,f) and diceCT segmentation (Fig. 2d,g), evident from the linear regressions with a slope substantially lower than 1 (0.39 for polygonal modelling, adjusted R^2^ = 0.6977, and 0.32 for diceCT-based, adjusted R^2^ = 0.6967). The diceCT-based muscles potentially ‘underperformed’ (Fig. 2d,g) as the tendons could not be segmented and the resulting volumes, and therefore masses, were thus smaller. In smaller muscles (e.g., IT1), the polygonal model-reconstructed muscle fell close to the median of the observed data (between *Q*_1_ and *Q*_3_, Fig. 2a), whereas the diceCT-segmented muscle was just within the range of observations. Another reason why the iodine-stained segmentation underperformed could be that the muscles may have undergone volume shrinkage (dehydration) during fixation^21,89–92^ and may, therefore, have been smaller than expected from the physical measurements. The polygonally modelled muscles, which included the tendons in the volume calculations, however, ignored the ~ 5% higher density of the tendons (1120 kg/m^3^;^93^), which potentially negatively influenced their muscle mass estimates as well. Partitioning muscle segments into muscle and tendon is not straightforward for extant taxa and particularly difficult for extinct taxa, for which no muscle architectural information is directly preserved. However, the density difference affects only a small part of the total volumes and is thus almost negligible. A correction factor could address this issue, which, however, might not be uniformly applicable to all taxa without assuming musculoskeletal isometric scaling and could thus potentially be problematic in itself. It is, nonetheless, very encouraging that the muscle mass estimates of the *iterative polygonal modelling approach* generally matched the masses obtained from the iodine-stained CT data (Fig. 2b,e,h) and thus indicate reliable results. Especially, as Bribiesca-Contreras and Sellers^21^ generally found robust agreement between volume calculations of diceCT segmented muscles and physical measurements of those same muscles.

The LoA calculations based on the modelled muscles realistically described the LoA on the path from muscle origin to insertion (i.e., Fig. 3c,d) in most cases. In a specific and limited case, the LoA calculations slightly deviated from the presumed actual LoA, such as the kink in the LoA of the *M. deltoideus spinalis* (Fig. 3d). This discrepancy could be directly attributed to a limitation of the way the LoAs are calculated. Due to the wide attachment area, which is angled in relation to the long axis of the muscle, and a single vector along the muscle long axis describing the cut axis, the muscle was not sliced parallel to the origin surface but at an angle incorporating the medial part of the attachment into the slices. This artificially dragged the slice centroids towards the medial side (see Supplementary Figure S6). Therefore, our LoA estimation script might result in slight deviations of the LoA close to the attachment site for muscles with sheath-like and wide attachment areas; a similar limitation was discussed by Allen et al*.*^73^. However, these deviations should not directly affect LoA-dependent measurements (i.e., moment arms) as the discrepancy is regionally contained (close to the attachment site) and the LoA can be corrected in other software packages used for LoA-dependent measurements such as OpenSim^53,54,70–72,74,76,94^, GaitSym^47,50,95–97^ or Simm^52,98–102^. Therefore, the *iterative polygonal modelling* approach offers an intuitive way to streamline all muscle modelling steps in which: (1) during dissection of a species to collect parametrical information, the researcher can scan each muscle layer to produce 3D models of each layer, from which (2) the muscle layers can directly be used to guide 3D modelling of each specific muscle of interest, after which (3) parametrical information from dissection and the 3D muscle model can be combined to produce a specified LoA per muscle in musculoskeletal modelling software (e.g., GaitSym, OpenSim, Simm), complete with subject-specific muscle parameters. This may then minimise potential modelling errors, which may be apparent in other studies. For example, in Wiseman et al*.*^76^ different specimens of the same species each provided independent information (i.e., one specimen was dissected, whereas a different specimen provided muscle paths via diceCT-based scanning). This information then had to be scaled accordingly to produce a singular musculoskeletal model.

In conclusion, the workflow presented herein and evaluated using data from crocodylians and *Gorilla* enables the reconstruction of crucial missing muscle parameters for biomechanical models and simulations of living and extinct animals with more confidence and allows appraising values predicted through other methods. Overall our results show that the method yields reasonable estimates for muscle masses and sufficiently accurately describes LoAs in extant animals, and therefore should be directly applicable to extinct animals, as demonstrated for *Euparkeria*. We, therefore, advocate the suitability of *iterative polygonal modelling* guided via physical cross-sections (Fig. 5), or diceCT and/or MRI-scan slices to model musculature in extant and extinct species. This method circumvents the often prohibitive cost of many segmentation software packages^1^, which can be limiting to many researchers. Rather, this method is ready for use in Maya, which is free to all researchers/academics registered at an educational institution (schools, colleges, universities and home-school programs worldwide which are government accredited). Alternatively, the method is generally adaptable for use in freeware, such as Blender software (https://www.blender.org/). The wide applicability of our method will have great advantages for improving the development of subject-specific models. Additionally, our approach represents a valid and fast alternative to CT scanning of iodine-stained specimens (diceCT) for cases in which the latter is not feasible (e.g. costs or availability of materials, specimens and/or CT scanners; time-consuming nature of manual segmentation^1^).

## Material and methods

Several taxa were chosen as case studies to test and evaluate our methodology. No animals were harmed for the purposes of this study. In sum, our workflow pattern was structured as follows: to model the musculature of an extinct species (here, *Eupakeria capensis*), we first had to model the musculature of a comparative extant species (*Crocodylus niloticus*). We then statistically determined the efficacy of our procedure. Next, to determine if the method was adaptable to other forms of data capture, we modelled the musculature of another extant species (*Gorilla gorilla gorilla*) based upon data collected during dissection, thus streamlining the whole process. Each process is described in detail below and/or the Supplementary Information.

### Extant reptile: Nile crocodile

The Nile crocodile (*Crocodylus niloticus*) allowed us to evaluate our results with a maximally comparable dataset. Skeletal and muscle geometry were obtained from an iodine-stained and μCT scanned (diceCT^4^) hindlimb of a single juvenile female individual (see: Supplementary Information for scan parameters and further information). Hindlimb muscle measurements of various Nile crocodile specimens were obtained from Allen et al*.*^103^, Wiseman et al*.*^76^ and this study (see Supplementary Information for raw measurements).

### Extinct fossil reptile: *Euparkeria*

*Euparkeria capensis* is a small archosauriform from the Middle Triassic Burgersdorp Formation of South Africa^104^. Its osteology is well known from numerous specimens^104,105^ and it is a phylogenetically important taxon as a close relative of the last common ancestor of birds and crocodiles^105–107^. It is therefore a key taxon to investigate the ecology and ancestral locomotory capabilities of archosaurs^108–110^. Additionally, its limb morphology is similar to modern crocodilians^108^ and thus well suited as a case study to volumetrically reconstruct the hindlimb musculature of an extinct taxon. Multiple individuals from the Iziko South African Museum, Cape Town, South Africa (SAM) and University Museum of Zoology Cambridge, Cambridge, UK (UMZC) were μCT scanned in Stellenbosch, South Africa and Cambridge, UK (see Supplementary Information for scan parameters and further information) and scaled isometrically to the most-complete specimen (SAM PK 5867). The composite hindlimb skeleton and pelvic girdle was articulated in an osteologically feasible posture^110^ for muscle reconstruction.

### Extant mammal: Western lowland gorilla

The Western lowland gorilla (*Gorilla gorilla gorilla*) offered a unique opportunity to test the methodology on a single specimen for which a multitude of data was available. Surface scans capturing muscle orientation and attachment sites were obtained during the dissection that provided muscle architectural measurements using a structured-light surface scanner (Artec Space Spider with Artec Studio 12, Artec 3D, Luxembourg). The skeletal geometry was obtained from a medical CT scan performed at the Ohio State University College of Veterinary Medicine^94^; see Supplementary Information for further information. The female specimen died of old age and was donated by the Cleveland Metroparks Zoo to the Cleveland Museum of Natural History for research purposes.

### Polygonal muscle modelling approach

The fundamental basis for every muscle in our 3D modelling approach builds a closed cylinder, which is morphed to match the shape of the muscle, with 8, 12 or 16 faces describing its circumference, depending on the surface area of the muscle origin and muscle complexity, whereas the caps represent the origin and insertion respectively. The height (i.e., the long axis) of this cylinder can be arbitrarily and iteratively subdivided and adjusted to alter the circumference and shape of the cross-section at any given point, or to change the curvature and path of the modelled muscle to prevent it from intersecting with other muscles or bones. We used the software package Autodesk Maya 2019 in this study, which is freely available for educational/academic usage. Some other software packages, e.g. the open-source freeware Blender, allow for the same 3D modelling approach (see^1,111^ for further examples). The general workflow of the 3D muscle modelling may differ slightly based on the software package used; however, the workflow should be readily adaptable.

#### Iterative polygonal muscle modelling

This workflow allows the three-dimensional estimation of muscle volumes and paths for extant and extinct taxa of which 3D scans of bones have been acquired but no direct information on muscle volume is present. We developed the workflow based on the extant Nile crocodile (*Crocodylus niloticus*; 3D bone models were obtained from^76^; Fig. 1a,c) and the extinct stem-archosaur *Euparkeria capensis* (3D bone models were obtained from^110^; Fig. 1b,d). The muscle size was qualitatively constrained by physical cross-sections of *Alligator mississippiensis* hindlimbs (Fig. 5 and additional photographs of the cross-sections are provided in the Supplementary Information; Supplementary Figures S1–S3), which were used as guides along the thigh (for both the crocodile in our sample to test our method vs. diceCT data, and *Euparkeria*) and the shank (*Euparkeria* only). As alligators and crocodiles are both crocodylians and *Euparkeria* also had a similar limb morphology^108^, we used alligator cross-sections as constraints. For the general application of this method to other taxa, it should be suitable to use any closely related and/or morphologically similar taxon/taxa to constrain the musculature. We advise to use such tomographic data (physical, CT or MRI if available) of closely related taxa in combination with information derived from the EPB^53^ to guide the reconstruction of the missing musculature and constrain their extent and spatial organisation, which can be highly evolutionarily constrained in vertebrates^8,112–117^; but see^118^, thus addressing one of the major uncertainties in the modelling process, see^59^. The step-by-step guide of our polygonal muscle modelling approach is described in detail in the Supplementary Information.

#### Application to surface data (surface mesh retopology)

*Iterative polygonal modelling* is not limited to cross-sectional information, this method can be used in conjunction with muscle surface scan data that can (1) streamline processes in which the user can also collect dissection data at the same time as muscular configurations, and (2) further minimise costs and time commitment for the user because expensive CT-scan data after lengthy staining processes do not need to be collected^1^. However, it is not required to use surface scan-technology that can be expensive and prohibitive itself^85,87^; photogrammetry is an alternative possibility^84,86^, in which the user can further minimise total costs by even using a personal smartphone rather than an expensive camera or laser scanning^87^ to capture a series of photographs and then subsequently use freeware to build 3D models of each muscle layer (e.g.^1,83^).

We modelled individual muscle bellies of a Western lowland gorilla specimen through retopology^81,119,120^ of surface scan data that were collected during dissection of the shoulder musculature in combination with CT scan-derived bone models of the same specimen^94^ (see Supplementary Information for detailed step-by-step instructions). Such surface scans can be collected during the systematic removal of muscle ‘layers’ (i.e., from superficial to deep). However, in comparison to diceCT or MRI data, where the complete three-dimensional information of a muscle is characterised^4,26,28,121^, surface scan data represent a challenge. The third dimension (i.e., volume) of a muscle is only partially derived, because only the extent on the surface can directly be inferred. Combining surface data from multiple steps during a dissection (i.e., collecting surface scans via the systematic removal of individual muscles or layers from superficial to deep musculature), however, allows retroactive estimation of the 3D shape of the muscles by constraining their volume through the difference of two or more surface meshes. A polygonal retopology of the muscle surfaces allows fast quantification of the negative space between different dissection steps. The step-by-step guide of our workflow on surface data is described in detail in the Supplementary Information.

#### Muscle attachment centroid calculation

The centroids of the attachment areas can either be estimated for extinct taxa^44,50,51,53,60,97,122–124^ or measured/inferred from digitized attachment areas of extant taxa^69,73,74,78,79,88,125^. Additionally, we developed a code (Supplementary Information) to streamline musculoskeletal modelling workflows that now allows to directly calculate the attachment centroids and the attachment area for extinct and extant taxa from the vertices placed on the attachment area during the *iterative polygonal muscle modelling* approach presented herein (see Supplementary Information for muscle attachment definition) or for selected faces of a segmented muscle (i.e., diceCT or MRI) or selected faces of a muscle scar/attachment area on a bone, as follows.

The centroids were calculated by selecting the faces of the attachment area and running the *Maya polygon surface centroid calculation* script written in Maya Embedded Language (MEL; code provided in Supplementary Information). The script triangulates the muscle attachment areas to subdivide faces with more than three vertices into individual triangles to facilitate the calculation of their centroid. The area of each triangle can be described using *Heron’s Formula* and the distances between the 3D coordinates of each vertex (i.e., $a = \left| {\overrightarrow {BC} } \right|, b = \left| {\overrightarrow {AC} } \right|, c = \left| {\overrightarrow {AB} } \right|$), where the semi-perimeter *s* is calculated as:

1 $$s = \frac{a + b + c}{2}$$

And its area *w*_*i*_ is calculated as:

2 $$w_{i} = \sqrt {s\left( {s - a} \right)\left( {s - b} \right)\left( {s - c} \right)} { }$$

The centroid of each individual triangle *C*_*i*_ is calculated by averaging the position of each vertex:

3 $$C_{i} = \frac{A + B + C}{3}{ }$$

The centroid of the attachment area *C*_*A*_ is then calculated by weighting the centroid of each triangle *C*_*i*_ by their respective face area *w*_*i*_ in comparison to the total area *W*_*A*_ of the attachment area:

4 $$W_{A} = \mathop \sum \limits_{i = 1}^{n - 2} w_{i}$$

and, therefore:

5 $$C_{A} = \frac{1}{{W_{A} }}\mathop \sum \limits_{i = 1}^{n - 2} w_{i} C_{i} ,{ }$$

where *n* is the number of vertices describing the attachment area (i.e., the number of vertices projected onto the bone during modelling or describing the selected faces).

#### Muscle line of action estimation

The *Maya muscle line of action estimation* MEL script (provided in the Supplementary Information) was inspired by Allen et al*.*^73^. However, this previous workflow requires several software packages and processing steps. Our process offers an intuitively fast and straightforward way to automate the estimation of a single LoA for downstream analyses in a single software package (Maya). This process depends on four input parameters: the 3D coordinates of the origin and insertion centroids, the 3D model of the muscle (path) and the number of slices (user-specified; see below). The attachment centroid positions are best represented as *Locators*, i.e., as calculated from faces (using the *Maya polygon surface centroid calculation* script above) or imported from a different source (e.g. see^71,101–105^). The script slices a muscle into a user-specified number of slices along an axis from the origin to the insertion (i.e., the 3D coordinates of the respective locators). Our process (see Supplementary Information) calculates the centroid of each slice in the same fashion as the *Maya muscle attachment centroid calculation* MEL script (i.e., by subdividing the slices into triangles and then weighting their centroids by their respective area). The centroids of all slices are then threaded together to form a path from origin to insertion representing the LoA. This path is then converted into a NURBS curve and subsequently into a polygonal object for visualisation (Figs. 3c,d, 4a) that can subsequently be exported for musculoskeletal modelling and simulation software, such as OpenSim^71^ (Fig. 4b). This method has the potential to streamline the workflow of prior studies (e.g.^73,74,76,94^) or to improve the modelling of LoA dependent measurements (e.g.^46,70,72^). The LoA estimation code is written in Maya’s native programming language, i.e., Maya Embeded Language (MEL), and is thus conceptualized only for Maya. Therefore, it is not easily adaptable for other software packages without major rewriting of the code provided herein, as variable definitions and/or specific functions may differ depending on the software package; yet the approach has transferrable concepts.

### Statistical analysis of muscle mass comparisons in the Nile crocodile

The volumetric muscle models created using the *iterative polygonal modelling approach* (either surface-based or tomography-guided) can be evaluated by the criterion that simplifications and/or predictions should quantitatively fall within the range of physical measurements or follow observed patterns^21,29,33^. The 3D muscle masses of both the diceCT segmented and the reconstructed muscles using the *iterative polygonal modelling approach* were calculated from their volumes with an assumed homogeneous density of 1060 kg/m^3^ ^3,102,126,127^. These calculated muscle masses of the Nile crocodile were then compared to the median of the body-mass-normalised measurements of the respective muscles of other Nile crocodile specimens (Fig. 2a; Table 1). Discrepancies between the actual muscle masses and the 3D representations of the muscles had to be interpreted broadly, because no direct comparison with the actual muscle mass was possible. As direct, dissection-based measurements were not available for the Nile crocodile specimen used in this study, our model-estimated muscle masses were compared to a broader set of published data on Nile crocodile muscle masses^76,103^ as well as muscle volumes calculated from the segmented iodine-stained (diceCT) muscles of the same specimen (Wiseman et al*.*^76^ and this study; see Supplementary Information). Therefore, we performed a Bland–Altman analysis^128^ using R software^129^ with the R package ‘blandr’^130^ to compare the different measurements and muscle mass estimates, and their agreement. Additionally, Mann–Whitney U tests were performed between the different methods to test for differences in the muscle mass estimates.

## Supplementary Information

Supplementary Information 1.

Supplementary Information 2.

## Data Availability

The data that support the findings of this study are available in the article and/or Supporting Information and under the following link https://doi.org/10.6084/m9.figshare.16903897. All code used in this study to replicate our methodology and findings is available in the Supporting Information and under the following link https://doi.org/10.6084/m9.figshare.16903900. All muscle 3D models to replicate our findings are available under the following link https://doi.org/10.6084/m9.figshare.16903894; additional 3D models (bones and/or surface scans) are available from the corresponding author upon request.
