# Supplementary material for: Three-dimensional polygonal muscle modelling and line of action estimation in living and extinct taxa
Source: Sci Rep. 2022 Mar 1;12:3358. doi: 10.1038/s41598-022-07074-x (PMC8888607; doi:10.1038/s41598-022-07074-x)
Supplement: Supplementary file 1 — Supplementary Information 1. [file 41598_2022_7074_MOESM1_ESM.pdf]

SUPPLEMENTARY INFORMATION

for

Three-dimensional polygonal muscle modelling and line of action estimation in living and extinct taxa

Oliver E. Demuth[1,2,*], Ashleigh L. A. Wiseman[1,3], Julia van Beesel[4], Heinrich Mallison[5,6], John R. Hutchinson[1]

[1] Structure & Motion Laboratory, Department of Comparative Biomedical Sciences, The Royal Veterinary College, Hatfield, UK

[2] Department of Earth Sciences, University of Cambridge, Cambridge, UK

[3] McDonald Institute for Archaeological Research, University of Cambridge, Cambridge, UK

[4] Department of Human Evolution, Max-Planck-Institute for Evolutionary Anthropology, Leipzig, Germany

[5] Centrum für Naturkunde (CeNak), University of Hamburg, Hamburg, Germany

[6] Palaeo3D, Rain am Lech, Germany

*corresponding author: oed24@cam.ac.uk

CONTENT

Supplementary Information S1 **3**

Specimen information **3**

Nile crocodile. 3

Euparkeria capensis. 3

Western lowland gorilla. 4

CT scan parameters **4**

Supplementary Figures **5**

Supplementary Method S1. Step-by-step workflow description of iterative polygonal muscle modelling **15**

Supplementary Method S2. Step-by-step workflow description of application of iterative polygonal muscle modelling to surface data **18**

Supplementary Method S3. ***MAYA polygon surface centroid calculation*** **MEL script** **20**

Supplementary Method S4. ***MAYA muscle line of action estimation*** **MEL script** **23**

Supplementary References **29**

Supplementary Information S1

Specimen information

Nile crocodile.

All animal data were collected as part of previous studies [1,2] at the Royal Veterinary College, UK under the Home Office (United Kingdom) project license P0806ABAD and approval of the College's Ethics and Welfare Committee (approval number 2016-0089 N). After euthanasia and following standard ethical procedure using anaesthetic overdose [1,2], a Nile crocodile (*Crocodilus niloticus*; specimen DDNC03; Wiseman et al. 2021) was stained with Lugol's solution [3], 4% iodine with 10% neutral-buffered formalin, for 93 days and then microCT scanned at the University Museum of Zoology Cambridge, UK using a Nikon XTEK XTH 225 ST scanner (see CT scan parameters below). The bone and muscle volumes were manually segmented and extracted in MATERIALISE MIMICS 22.0 (https://www.materialise.com/en/medical/mimics-innovation-suite).

Euparkeria capensis.

CT data of several specimens of the Triassic stem-archosaur *Euparkeria capensis* from the Iziko South African Museum (SAM) and the University Museum of Zoology Cambridge (UMZC) were obtained from a previous study (Demuth et al., 2020; see CT scan parameters below). The bones were segmented in AVIZO 9.7 Lite (https://www.thermofisher.com/uk/en/home/electron-microscopy/products/software-em-3d-vis/avizo-software.html) and combined into a composite hindlimb with elements from the individual specimens scaled isometrically to the most complete specimen (SAM PK 5867) and articulated in an osteologically feasible posture [4].

Western lowland gorilla.

A single western lowland gorilla (*Gorilla gorilla gorilla*) specimen was used in this study. The specimen, published on previously [5], was a zoo animal, which was euthanized at the age of 48.8 years in December 2012 after suffering from various age-related ailments, and was subsequently ethically acquired through the Cleveland Museum of Natural History, Erie Zoo and Cleveland Metroparks Zoo [5]. The CT scan to extract the bone models was conducted at the Ohio State University College of Veterinary Medicine using a *Revolution Evo Lightspeed* (see CT scan parameters below). The shoulder and upper arm bone geometries were manually segmented and extracted from the CT data using AVIZO 9.3 (https://www.thermofisher.com/uk/en/home/electron-microscopy/products/software-em-3d-vis/avizo-software.html).

CT scan parameters

TABLE S1. CT scan settings and scan resolutions for all datasets. *Gorilla gorilla gorilla*, *Crocodilus niloticus* (DDNC03) and *Euparkeria capensis* (prefix ID-codes: SAM and UMZC). The scanned blocks of both SAM PK 5867 and SAM PK 6047A are the ones containing the pelvis. UMZC T.692 1, 2 and 3 correspond to ankle bones scan 1 and 2 and the scan of the foot block.

Specimen	Voltage (kV)	Current (μA)	Exposure duration (ms)	Voxel size (μm)	Number of slices	Resolution (pixels)
Gorilla	120	319	600	625-977	1843	512x512
**DDNC03**	200	200	708	92-125	1998	738 x 1999
**SAM PK 5867**	170	400	500	90	1792	1387 × 515
**SAM PK 6047A**	170	400	500	90	1618	673 × 971
**SAM PK K8309**	170	400	500	50	1617	1206 × 550
**UMZC T.692 1**	115	120	1000	200	1568	1266 × 1197
**UMZC T.692 2**	115	120	1000	200	1799	1447 × 1301
**UMZC T.692 3**	190	170	1415	200	1999	1418 × 2000

Supplementary Figures

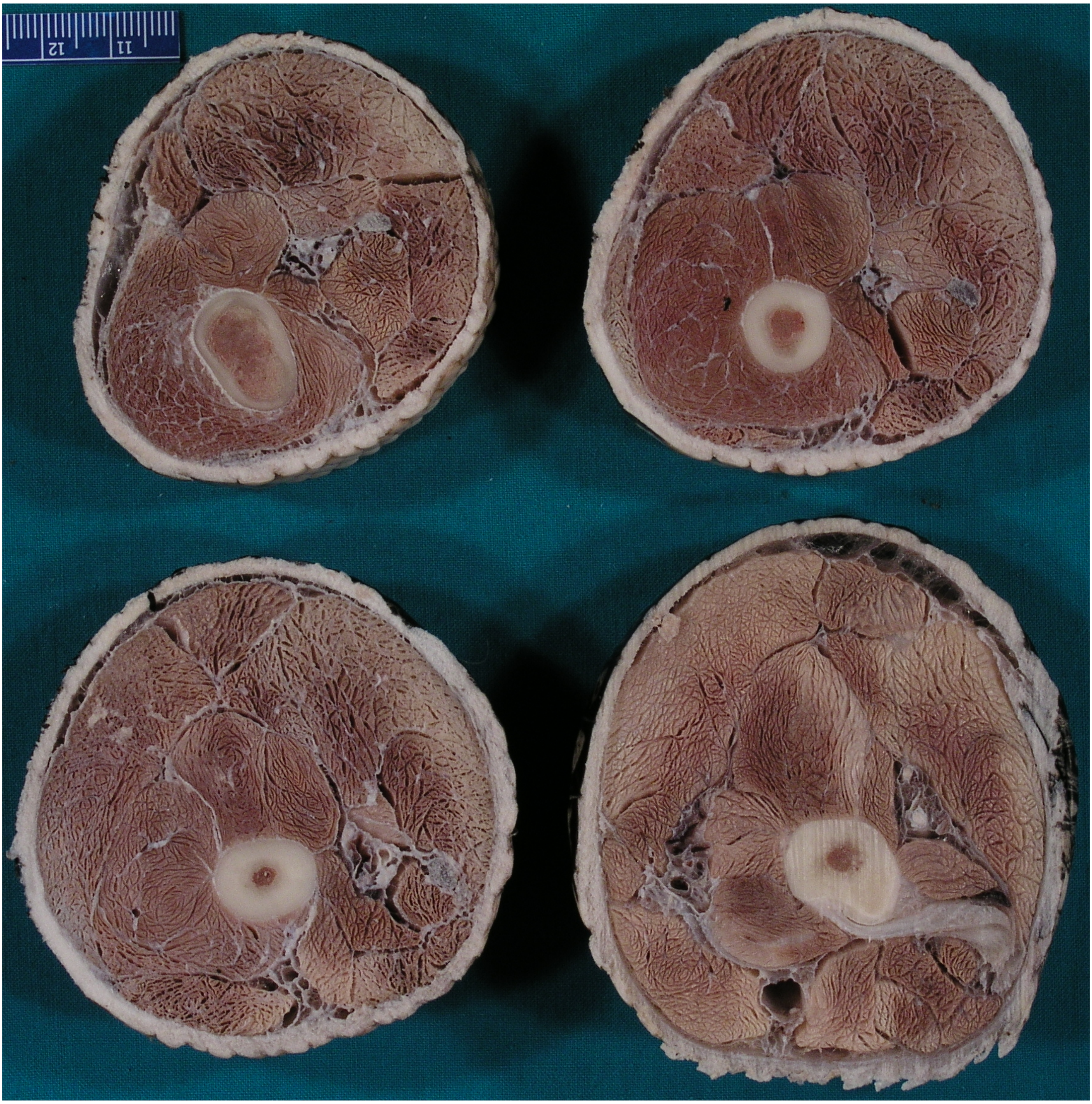

Figure S1. Slices through a right *Alligator* thigh. Slices follow from the lower right corner in a Z-like fashion from proximal to distal. Scale bar in cm. For labelled muscles, please see Figure 2.

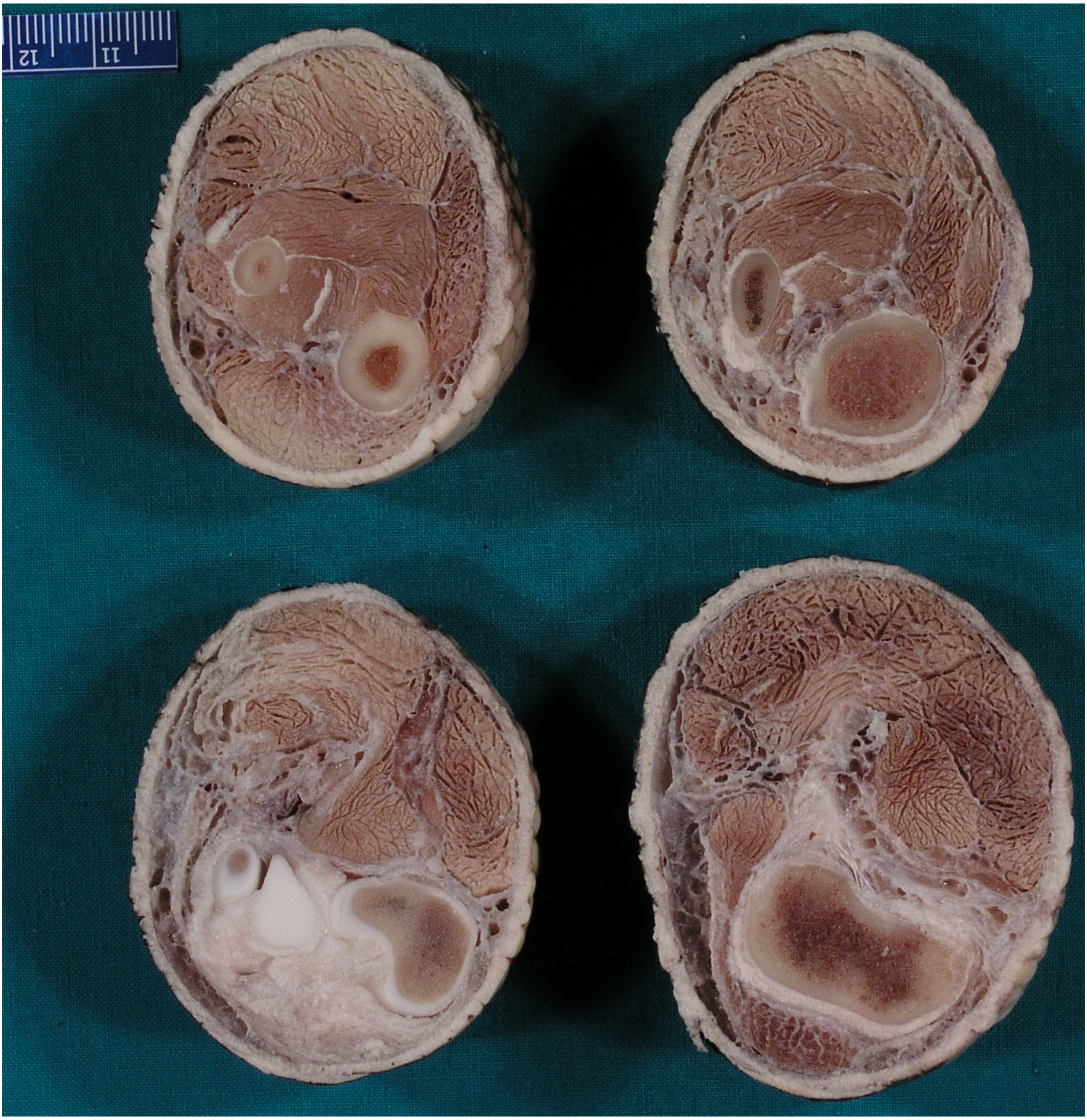

Figure S2. Slices through a right *Alligator* knee and proximal shank. Slices follow from the lower right corner in a Z-like fashion from proximal to distal. Scale bar in cm.

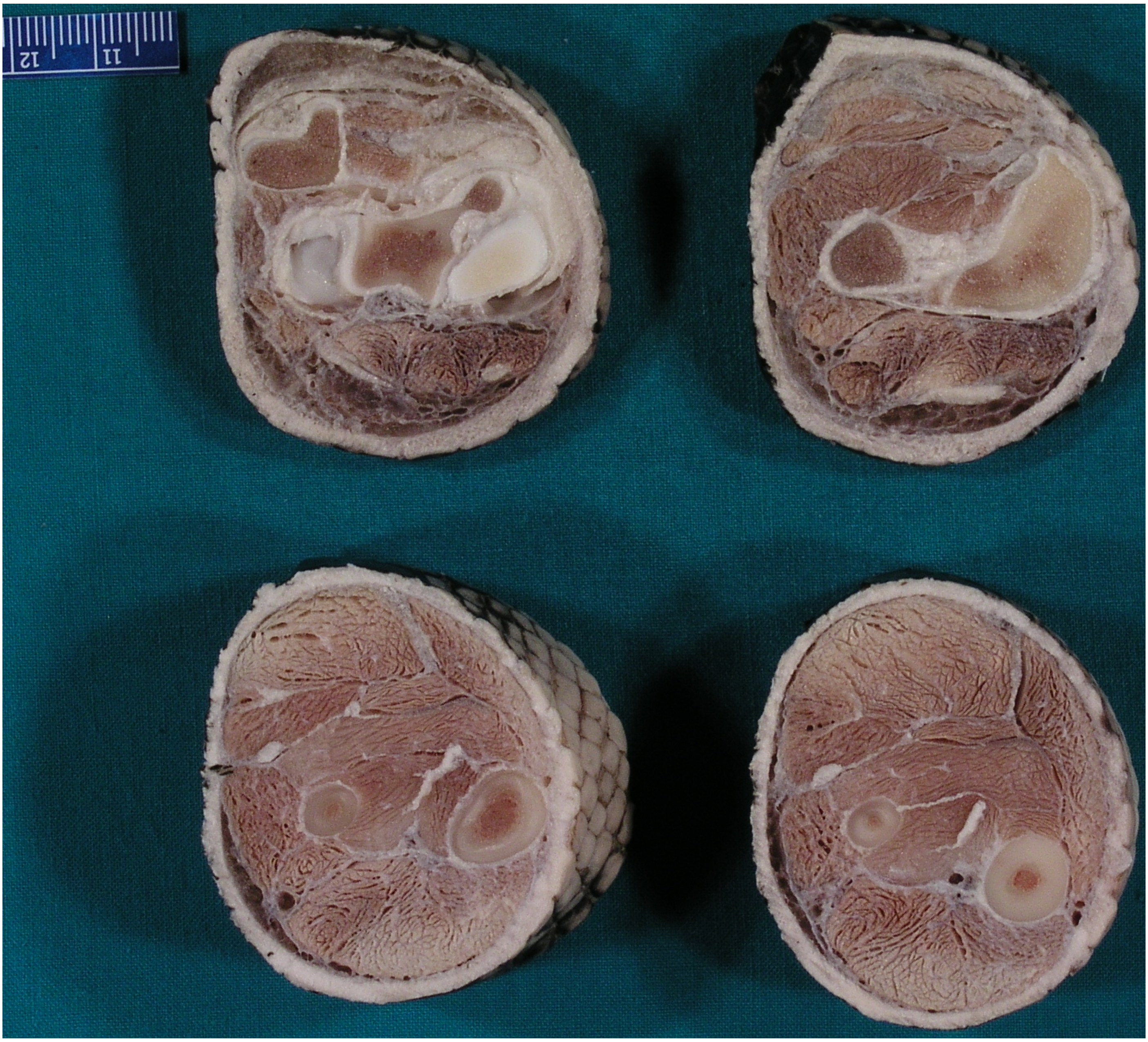

Figure S3. Slices through a right *Alligator* shank and ankle. Slices follow from the lower right corner in a Z-like fashion from proximal to distal. Scale bar in cm.

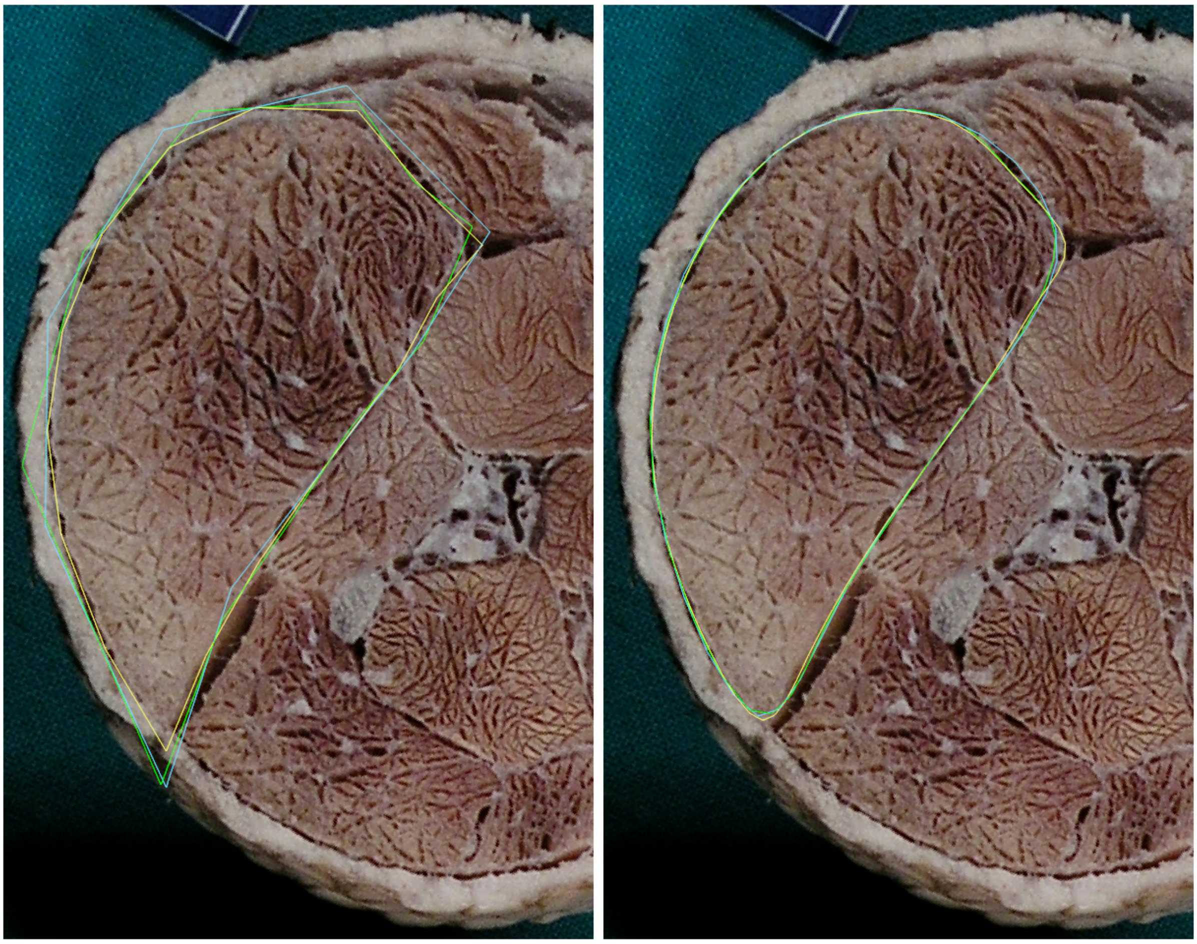

Figure S4. Comparison between different numbers of vertices used to describe the *M. flexor tibialis externus* (FTE) muscle belly. Left before and right after smooth preview using the OpenSubdiv Catmull-Clark algorithm [6]. Blue, 8 vertices; green, 12 vertices; yellow 16 vertices.

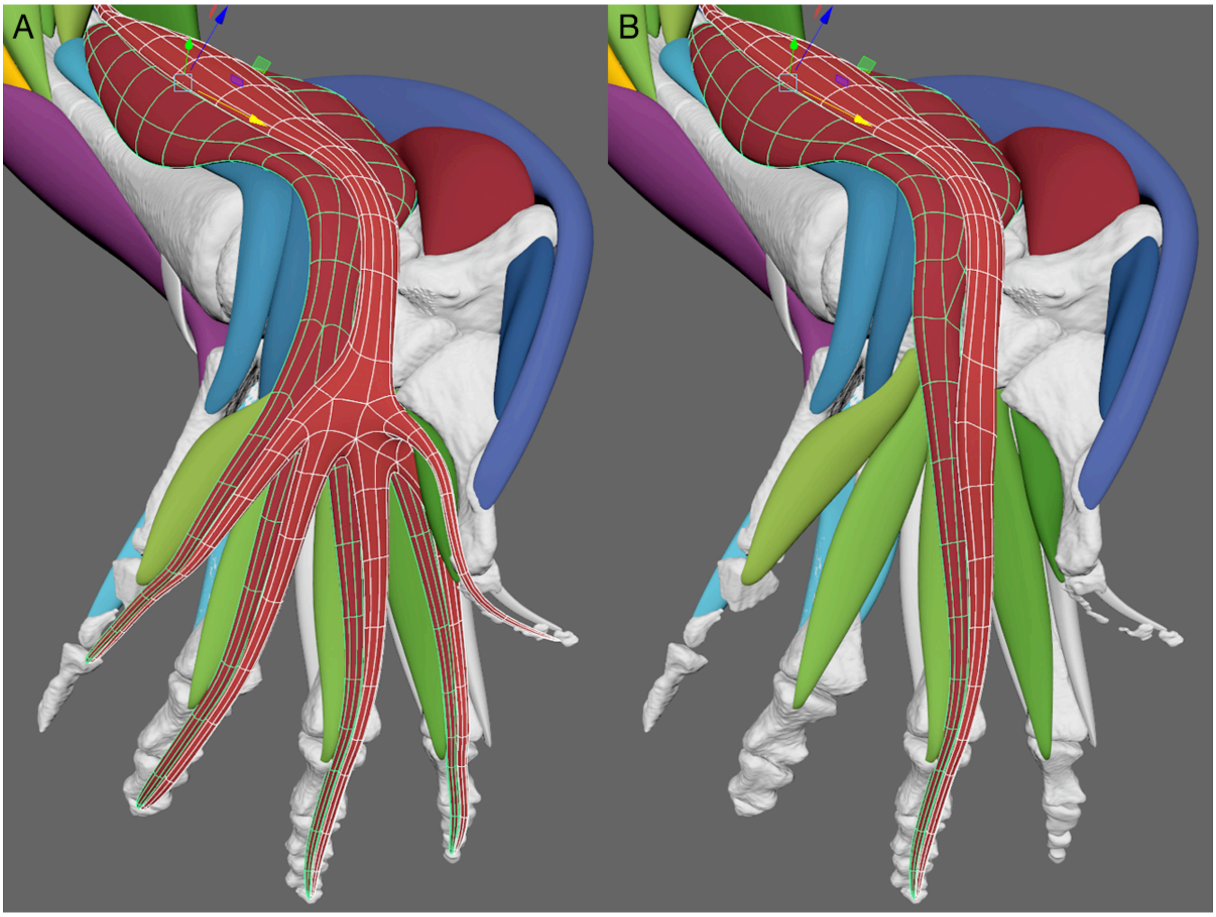

Figure S5. Trimming of tendons for LoA estimation for *Euparkeria*. The additional tendons for *M. flexor digitorum longus* (green face outlines) and *M. flexor hallucis longus* (white face outlines) before (A) and after (B) trimming of the tendons. Only the tendon to the third digit has been retained for LoA estimation for musculoskeletal simulations, otherwise the LoA would move erratically across the foot between the different tendons. If desired the LoA could be estimated for each individual tendon through removal of the other tendons and computation of the *MAYA muscle line of action estimation* script for each case. Superficial muscles have been removed to show the relevant muscles.

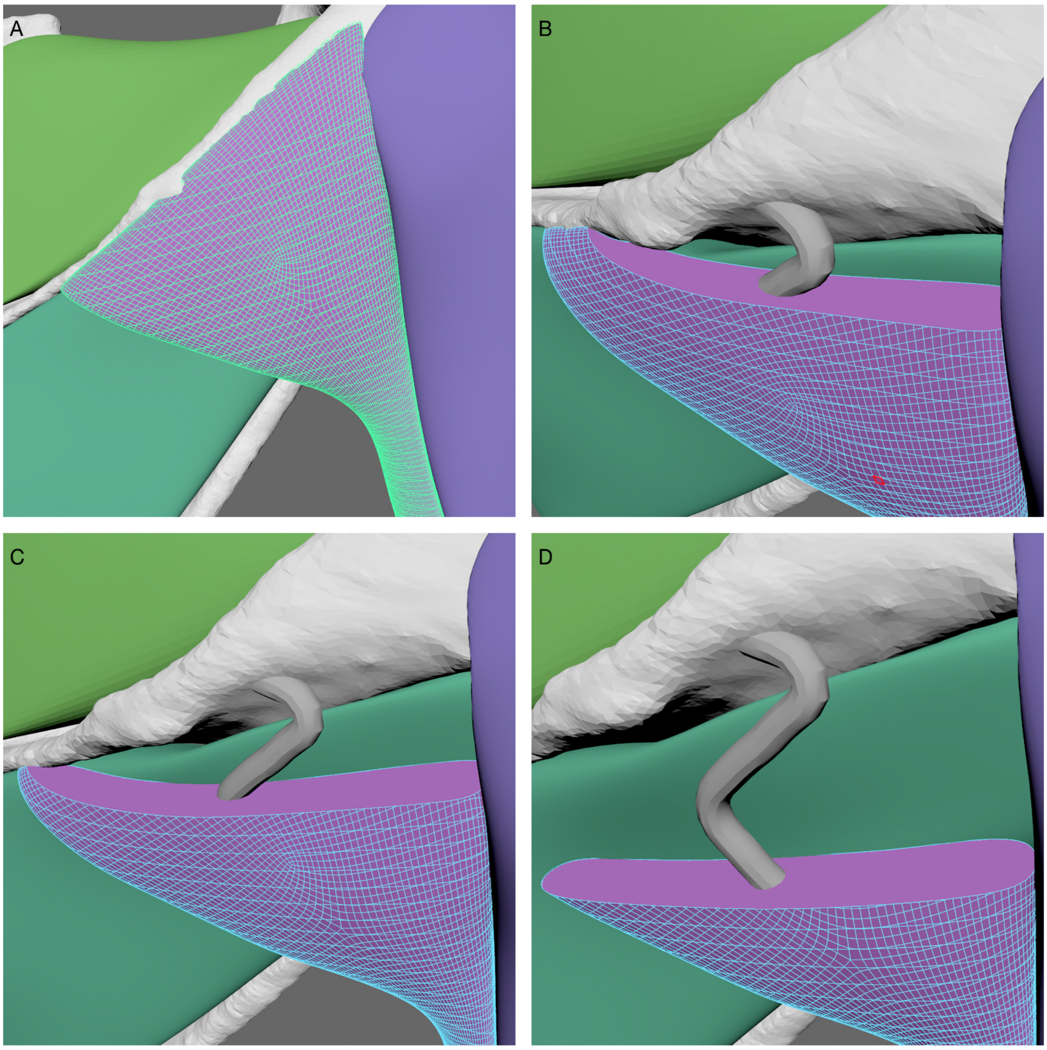

Figure S6. Line of action artefact of the *M. deltoideus spinalis* in *Gorilla*. Due to wide and sheath-like attachment on the scapula the muscle slices incorporated the attachment area (A) and thus dragged the slice centroids medially (B,C) until the attachment was no longer part of the slices (D).

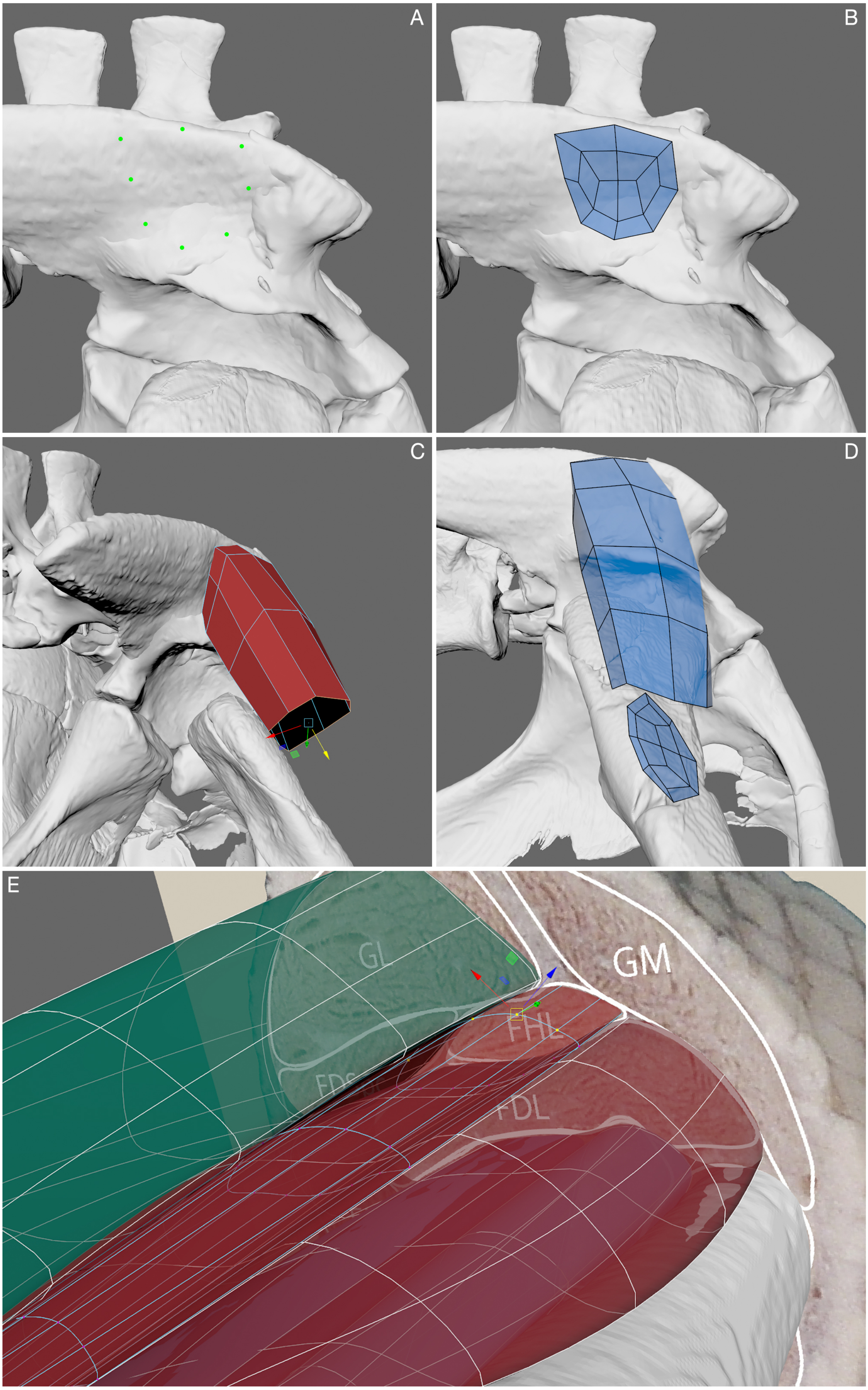
A
B
C
D
E
GL
GM
FHL
FDL

Figure S7. *Iterative polygonal muscle modelling* workflow. (A) Placement of the individual vertices describing the muscle attachment surface of the *M. iliofemoralis* (IF) of *Euparkeria* onto the 'live' ilium in the *Modelling Toolkit* in MAYA. (B) Creation of the origin attachment surface through connection of the vertices with faces in the *Modelling Toolkit*. (C) Extrusion of the edge loops to build the muscle belly. (D) Creation of the insertion on the 'live' femur in the *Modelling Toolkit*. (E) Iterative adjustment of the vertices of a muscle belly in comparison with the surrounding muscle bellies and the alligator lower hindlimb cross-sections. Note that the smooth muscle meshes are displayed using the OpenSubdiv smooth preview and thus still retain the flexibility of the low number of vertices (as highlighted on the *M. flexor hallucis longus*, FHL). Note that the *Modelling Toolkit* in MAYA highlights the faces of an edited object in transparent blue (B, D) while in basic mesh editing only the selected components are highlighted, e.g. the selected edges in orange (C) or the selected vertices in yellow (E).

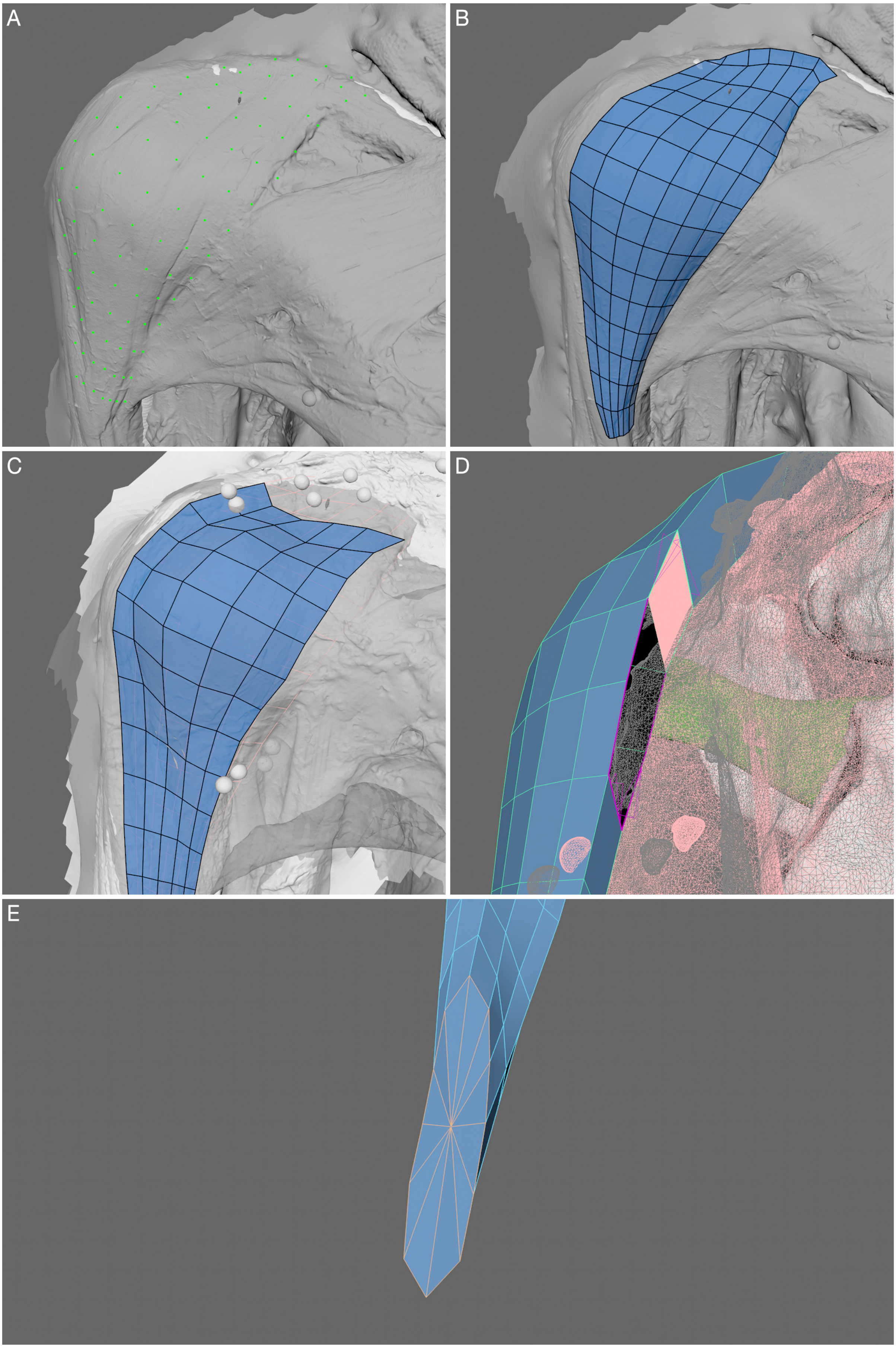
A
B
C
D
E

Figure S8. *Iterative polygonal muscle modelling* workflow for surface data. (A) Placement of the individual vertices onto the 'live' surface of the *M. deltoideus clavicularis* of the *Gorilla* specimen using the *Modelling Toolkit* in MAYA (frontal view). (B) Creation of the faces connecting the vertices to create a surface approximation (frontal view). (C) Placement of the vertices and faces on the surface scan after removal of the *M. deltoideus clavicularis* to capture the negative space (frontal view). (D) Combination of both surfaces using the *Append to Polygon* mesh tool to create a closed 'cylinder' from origin to insertion (oblique medial view). Note the face to be created is highlighted in pink. The surface meshes have been made semi-transparent (shown as grey and pink triangle edges) to improve the visibility of the mesh-closing process. The *M. supraspinatus* is visible in the background (green surface). (E) In absence of direct bone attachment information edges can be collapsed at the estimated attachment area to create a watertight mesh.

Supplementary Method S1. Step-by-step workflow description of iterative polygonal muscle modelling

1. After the origin(s) and insertion(s) were determined for a muscle, i.e., either through dissection, muscle scars and/or the EPB for extinct taxa; e.g., see [7–13], the bone where the muscle attaches to was converted into a 'live surface' [14]. This allowed drawing individual vertices directly onto the bone surface using the *Modelling Toolkit* in Maya. This is approach is frequently used in the 3D modelling industry to remesh or retopologise an existing model to get a more uniform topology [15–17]. Here, we used it to establish and define the 3D surface of the muscle attachment area. The vertices were arranged to uniformly surround the attachment area (Figure S7A). Depending on the complexity of this area, either 8, 12 or 16 vertices were used. This decision involves a trade-off between accuracy and complexity further downstream in the method. We concluded that 12 vertices are usually enough to define the outline of the muscle (see Figure S4), because they could reasonably track the tomographic muscle cross-sections, especially as the muscles are smoothed later on.
2. Additional vertices were added within the attachment area to refine the surface shape and make sure that the muscle surface accurately tracked the bone surface. The vertices were connected, and the faces were closed to produce the attachment surface of the muscle (Figure S7B).
3. In the next step, the live object was turned off to allow the individual vertices to be positioned independently from the surface of the bone within the 3D space; otherwise they would stay bound to the live object's surface. The edges surrounding the attachment area were extruded and the individual vertices repositioned to start shaping the muscle surface.

4. This edgeloop (i.e., the edges forming a closed loop perpendicular to the muscle long axis) was further extruded in a stepwise fashion towards the muscle's insertion or origin to start fleshing out the 3D shape of the muscle. Additional edgeloops were added where necessary to subdivide the faces and the muscle was adjusted to match the tomographic sections (Figure S7C). This was repeated until a rough shape of the muscle was obtained.
5. When the second bone attachment was reached (i.e., origin or insertion, depending on which direction the muscle was constructed from), the bone was converted into a live surface again and the vertices were placed onto the bone in the same manner as in steps 1 and 2 (Figure S7D).
6. Muscles with multiple heads and a common tendon (e.g. PIFE1-3 of Archosauria) should be modelled individually, i.e., each with an individual tendon attaching to the bone. Muscles with multiple insertions (e.g. *M. flexor digitorum longus* in the lower hindlimb of most Tetrapoda) should be modelled with multiple tendons, which can then be trimmed for the LoA estimation (see Figure S5). One of the advantages of polygonal modelling is that an additional tendon(s) can easily be extruded from polygonal faces, an option not readily available in NURBS modelling.
7. Steps 1 to 6 were repeated to model each individual muscle.
8. After two or more neighbouring muscles were completed, the positions of individual vertices of each muscle were re-examined and readjusted in an iterative manner to fill in any open spaces/gaps between the muscles as realistically as possible until an anatomically satisfactory result was achieved (Figure S7E). Additionally, the overall muscle shapes were examined to detect unrealistic shapes and modelling errors, such as double-bellied muscles (whereas a muscle has a thin part in the middle and bulges out proximally and distally) and corrected in comparison with the cross-sections.

9. After muscle construction, each muscle was smoothed, i.e., the faces were subdivided to obtain a higher resolution and more uniform curvature using the Maya inbuilt OpenSubdiv Catmull-Clark algorithm [6]. A division level of 2 or 3 was found to be sufficient. The number of faces grows exponentially with each division level, resulting in longer computational time further downstream with relatively little gain in fidelity.

Supplementary Method S2. Step-by-step workflow description of application of iterative polygonal muscle modelling to surface data

1. Before creating individual muscles, the superficial 3D surface scans captured during the dissection had to be aligned with the underlying surface scans and the bone reconstructions of the CT-scan data. Several discrete, homologous landmarks in the different scans, which were labelled during the dissection using pins, were used to orient and translate the models into position; see [5].
2. Instead of converting the bone surface into a 'live surface', the superficial-most scan was made 'live'. Whilst in the *iterative muscle modelling* workflow (above), the caps (i.e., attachment area) of the cylinder were created using the *Modelling Toolkit*, in this surface retopology the sides of the cylinder were constructed. Individual vertices were placed on the scan surface from origin to insertion in evenly spaced rows and columns (Figure S8A). The number of columns depended on the assumed portion of the muscle visible. The cylinder representing the muscle should have between 8 and 16 faces, as in the iterative modelling workflow.
3. The vertices placed on the muscle surface were then connected through faces and relaxed to receive a more uniform distribution of the vertices, approximating the scan surface (Figure S8B).
4. After the visible surface of a muscle was captured, the superficial-most muscle mesh was hidden, and the subsequent layer of the musculature (i.e., in order from superficial to deep) was made visible and 'live'. The negative space that the now hidden muscle left behind was captured by placing vertices onto the now visible adjacent muscle and/or bone surfaces (Figure S8C). The rows of the vertices were matched to the ones previously placed on the outer surface, thus ensuring that the vertices formed loops around the muscle with its long axis subdivided into several rows.

5. Step 3 was repeated for the newly placed vertices.
6. After we were satisfied with the retopology of the muscle surface and its negative space, both meshes were combined and connected using the *Append to Polygon* mesh tool (Figure S8D).
7. If the bone was exposed in any of the lower surface models (e.g. *M. supraspinatus*) or if derived from CT data, the origin and insertion of said muscle was created identically to steps 1 and 2 in the *iterative muscle modelling* workflow (see above). However, if this process is repeated in future studies and in the case that the bone cannot directly be exposed and/or a surface scan cannot be aligned with CT scan-based bone models, the origins and insertions will have to be approximated based on the anatomical understanding of the animal/specimen in question. The proximal and distal edgeloops should be positioned at their estimated attachment areas, extruded and merged to their centre to create a 'watertight' 3D model of the muscle (Figure S8E).
8. After each muscle was reconstructed, the meshes were smoothed using the OpenSubdiv Catmull-Clark algorithm [6] in MAYA. As above, a division level of 2 or 3 was sufficient.

Supplementary Method S3. ***MAYA polygon surface centroid calculation*** **MEL script**

To calculate the centroid of a polygonal surface, or in this case the centroid(s) of the muscle attachment area(s), i.e. origin and insertion, select the faces describing the attachment(s) and run the following script.

```
/* Maya polygon surface centroid calculation

    This script creates a Locator at the position of each surface centroid based on the
    selected faces of any mesh surface. The centroid is calculated based on the weighted
    average of the face centroids.

    This script works for single or multiple surfaces. For example when the faces of only
    one muscle attachment area are selected or when the faces of both origin and insertion
    are selected.

    Written by Oliver Demuth 26.03.2021
    Last updated 19.08.2021 - Oliver Demuth

    Note, before running the script make sure to have all relevant faces of the polygon
    surfaces (e.g. the muscle attachments) selected.

*/

//=========================================================//

/************  CALCULATE SURFACE CENTROID(S)  **************/


// get object name and faces from selection

string $selection[] = `ls -sl`;
int $sizeSelection = `size($selection)`;

string $t[];
string $tempT[];
string $mT[];

for ($i = 0;$i<$sizeSelection;$i++)
{
    tokenize $selection[$i] "." $tempT;

    $t[2*$i] = $tempT[0];
    $t[2*$i +1] = $tempT[1];
}

tokenize $t[0] "|" $mT;


// create temporary object duplicate

int $sizeBuff = size($mT);
string $nCopy = $mT[$sizeBuff-1] + "_duplicate";

duplicate -n $nCopy $t[0];


// project selection onto object duplicate

string $copySel[];

for ($k = 0;$k<$sizeSelection;$k++)
{
    $copySel[$k] = $nCopy + "." + $t[2*$k +1];
}


// extracts object surfaces

string $nShape = $nCopy + "Shape";
```

```
polyChipOff -ch 1 -kft 1 -dup 1 -off 0 $copySel;
polySeparate -rs 1 -ch 1 $nShape;


// define and name object surfaces

string $sel[] = `ls -sl`;
int $sizeSel = size($sel);

string $att = $mT[1] + "_attachment";
string $orig = $mT[1] + "_origin";
string $ins = $mT[1] + "_insertion";

string $mAtt[];

if ($sizeSel < 3)
{
    rename $sel[1] $att;
    $mAtt[0] = $att;
}
else
{
    rename $sel[1] $orig;
    rename $sel[2] $ins;
    $mAtt[0] = $orig;
    $mAtt[1] = $ins;
}

select -clear;


// calculate the centroid for each object surface

for ($j = 0;$j<($sizeSel-1);$j++)
{

    // triangulate attachment for centroid calculation

    select $mAtt[$j];
    polyTriangulate $mAtt[$j];


    // get vertex and face IDs from triangulated attachment

    string $vertIndicesStr[] = `polyInfo -fv`;
    int $sliceFaceNum = `size($vertIndicesStr)`;


    // calculate area and center of each triangle of the attachment

    string $vertIndices[];
    float $s;
    float $attFaceArea[];
    float $attFacetotArea = 0;
    vector $attFaceCenter[];

    for ($l = 0;$l<$sliceFaceNum;$l++)
    {
        tokenizeList($vertIndicesStr[$l], $vertIndices);

        string $VtxA = (string)$mAtt[$j] + ".vtx[" + $vertIndices[2] + "]";
        string $VtxB = (string)$mAtt[$j] + ".vtx[" + $vertIndices[3] + "]";
        string $VtxC = (string)$mAtt[$j] + ".vtx[" + $vertIndices[4] + "]";


        // querry position of each vertex and calculate distances between them

        vector $VtxAPos = `xform -q -t -ws $VtxA`;
        vector $VtxBPos = `xform -q -t -ws $VtxB`;
        vector $VtxCPos = `xform -q -t -ws $VtxC`;

        float $DistA = mag ($VtxBPos-$VtxCPos);
        float $DistB = mag ($VtxAPos-$VtxCPos);
        float $DistC = mag ($VtxAPos-$VtxBPos);
```

```
        //calculate area and center position of each triangle

        $s = ($DistA + $DistB + $DistC)/2;
        $attFaceArea[$l] = sqrt($s * ($s-$DistA) * ($s-$DistB) * ($s-$DistC));
        $attFacetotArea += $attFaceArea[$l];

        $attFaceCenter[$l] = ($VtxAPos + $VtxBPos + $VtxCPos)/3;
    }

    // calculate attachment centeroid by weighting the center position of each triangle by their
respective face area (i.e. COM calculation)

    float $wPos[];
    float $wPosSum = 0;

    float $attPosSumX = 0;
    float $attPosSumY = 0;
    float $attPosSumZ = 0;

    vector $centerPos;

    for ($m = 0;$m<$sliceFaceNum;$m++)
    {
        // calculate weighting factor for each triangle

        $wPos[$m] = ($attFaceArea[$m] / $attFacetotArea);
        $attPosVec = $attFaceCenter[$m];


        // weight each triangle center by its area

        $attPosX[$m] = (($attPosVec.x) * $wPos[$m]);
        $attPosY[$m] = (($attPosVec.y) * $wPos[$m]);
        $attPosZ[$m] = (($attPosVec.z) * $wPos[$m]);


        // caluclate X, Y, Z coordinates of slice center

        $attPosSumX += $attPosX[$m];
        $attPosSumY += $attPosY[$m];
        $attPosSumZ += $attPosZ[$m];

        $wPosSum += $wPos[$m];
    }

    select -clear;
    select $selection;


    // set center position for attachment $j and create Locator

    string $attLoc = $mAtt[$j] + "_LOC";

    spaceLocator - p 0 0 0 -n $attLoc;
    select $attLoc;
    move -r $attPosSumX $attPosSumY $attPosSumZ;
    select -clear;

}

// clean up

delete $nCopy;
clear $copySel;
clear $mAtt;
```

Supplementary Method S4. *MAYA muscle line of action estimation* MEL script

To calculate the line of action (LoA) of a muscle, select the origin and insertion locators (as calculated from the Method S1. *MAYA polygon surface centroid calculation* MEL script or imported from another dataset) and the muscle mesh and execute the following MEL script in the *Script Editor* in MAYA. The number of slices and the radius of the LoA can be specified in line 24 and 28, respectively. Additionally, there is an option to place Locators at the muscle slice centroids in line 32.

```
/* Maya muscle line of action (LOA) estimation

    This script creates a polygonal LOA for a muscle based on a user specified number of
    slices. The muscle is cut into a number of equidistant slices from origin to insertion.
    The centre of mass (COM), through which the LOA passes, is then calculated for each
    slice. A curve from origin to insertion through each slices' COM is created and
    converted into a polygonal mesh.

    Written by Oliver Demuth 19.02.2021
    Last updated 26.07.2021 - Oliver Demuth

    Note, before running the script make sure to have a locator for the origin and insertion
    at their respective world position as well as the muscle mesh selected.
    The script can take several minutes to run (depending on muscle mesh resolution).

*/

//=========================================================//

/******************  SET VARIABLES BELOW  ******************/

// Set the number of slices the muscle is cut into

int $numberOfDivisions = 50;

// Set radius of the line of action

float $LOARadius = 0.2;

// Do you want to place locators at the centroid of each slice?

int $createLOCs = 0; // 0 = no; 1 = yes


//=========================================================//

/************  SELECT 1. ORIGIN, 2. INSERTION,  ************/
/************   3. MUSCLE MESH AND RUN SCRIPT   ************/
/************      NO CHANGES NEEDED BELOW      ************/


//=========================================================//

/***************  CUTS MUSCLE INTO SLICES  *****************/


// get objects and their position

string $selection[] = `ls -sl`;
$origin = $selection[0];
$insertion = $selection[1];
$muscle = $selection[2];

string $mT[];
tokenize $muscle "|" $mT;

int $sizeBuff = size($mT);
string $nClean = $mT[$sizeBuff-1];
```

```
string $nCopy = $nClean + "_duplicate";
duplicate -n $nCopy $muscle;

vector $oPos = `xform -q -t -ws $origin`;
vector $iPos = `xform -q -t -ws $insertion`;


// get closest points on mesh to attachments

string $cPOMo = `createNode closestPointOnMesh`;
connectAttr -f ($muscle + ".outMesh") ($cPOMo + ".inMesh");
connectAttr -f ($origin + ".translate") ($cPOMo + ".inPosition");

string $cPOMi = `createNode closestPointOnMesh`;
connectAttr -f ($muscle + ".outMesh") ($cPOMi + ".inMesh");
connectAttr -f ($insertion + ".translate") ($cPOMi + ".inPosition");

vector $cPOMoPOS = `getAttr ($cPOMo + ".position")`;
vector $cPOMiPOS = `getAttr ($cPOMi + ".position")`;


// calculate vector from origin to insertion

vector $anlgleOffset  = $oPos - $iPos;
vector $offset = $cPOMoPOS - $cPOMiPOS;
vector $delta = $offset/$numberOfDivisions;


// calculate direction to cut the muscle into equidistant slices

vector $cutAim = `angleBetween -euler -v1 0 0 1 -v2 ($anlgleOffset.x) ($anlgleOffset.y)
($anlgleOffset.z)`;

vector $centerPos;
vector $sliceCenterPos[];
$sliceCenterPos[0] = $oPos;
$sliceCenterPos[$numberOfDivisions] = $iPos;

select -clear;


// create locator group if locators are to be created

if ($createLOCs > 0)
{
    string $tempLoc;
    string $centroidGRP;
    $centroidGRP = $nClean + "_LOC_GRP";
    group -em -n $centroidGRP;
    $createLOCs = 1;
}
else
{
    $createLOCs = 0;
}


// set progressBar

progressBar -edit
    -beginProgress
    -isInterruptable true
    -status "Estimating Line of Action"
    -maxValue $numberOfDivisions
    $gMainProgressBar;


// loop to slice muscle into specified number of divisions and calculate the center position of
each slice

for ($i = 1;$i<$numberOfDivisions;$i++)
{
    int $numFace[] = `polyEvaluate -f $muscle`;
    int $numMF = $numFace[0] - 1;
    string $mF = (string)$muscle + ".f[0:" + $numMF + "]";
```

```
    // cut muscle into slices

    polyCut
            -pc (($cPOMoPOS.x) - $i*($delta.x)) (($cPOMoPOS.y) - $i*($delta.y)) (($cPOMoPOS.z) -
$i*($delta.z))
            -ro ($cutAim.x) ($cutAim.y) ($cutAim.z)
            -ps 2 2
            -ef 1
            -eo 0 0 0
            $mF;

    select $muscle;

    polyCloseBorder;

    int $numSlice[] = `polyEvaluate -f $muscle`;
    int $numSF = $numSlice[0] - 2;
    string $nF = (string)$muscle + ".f[" + $numSF + "]";


    // selects newly generated face (slice of muscle at cut $i) and triangulate face

    resetPolySelectConstraint;
    selectType -pf true;
    select $nF;
    polyTriangulate $nF;


    // get vertex and face IDs from triangulated slice

    string $vertIndicesStr[] = `polyInfo -fv`;
    int $sliceFaceNum = `size($vertIndicesStr)`;


    // calculate area and center of each triangle of the slice

    string $vertIndices[];
    float $s;
    float $sliceFaceArea[];
    float $sliceFacetotArea = 0;
    vector $sliceFaceCenter[];

    for ($l = 0;$l<$sliceFaceNum;$l++)
    {
        tokenizeList($vertIndicesStr[$l], $vertIndices);

        string $VtxA = (string)$muscle + ".vtx[" + $vertIndices[2] + "]";
        string $VtxB = (string)$muscle + ".vtx[" + $vertIndices[3] + "]";
        string $VtxC = (string)$muscle + ".vtx[" + $vertIndices[4] + "]";


        // querry position of each vertex and calculate distance between them

        vector $VtxAPos = `xform -q -t -ws $VtxA`;
        vector $VtxBPos = `xform -q -t -ws $VtxB`;
        vector $VtxCPos = `xform -q -t -ws $VtxC`;

        float $DistA = mag ($VtxBPos-$VtxCPos);
        float $DistB = mag ($VtxAPos-$VtxCPos);
        float $DistC = mag ($VtxAPos-$VtxBPos);


        //calculate area and center position of each triangle

        $s = ($DistA + $DistB + $DistC)/2;
        $sliceFaceArea[$l] = sqrt($s * ($s-$DistA) * ($s-$DistB) * ($s-$DistC));
        $sliceFacetotArea += $sliceFaceArea[$l];

        $sliceFaceCenter[$l] = ($VtxAPos + $VtxBPos + $VtxCPos)/3;
    }


    // calculate slice center by weighting the center position of each triangle by their
respective face area (i.e. COM calculation)
```

```
    float $wPos[];
    float $wPosSum = 0;

    float $sfcPosSumX = 0;
    float $sfcPosSumY = 0;
    float $sfcPosSumZ = 0;

    vector $centerPos;

    for ($m = 0;$m<$sliceFaceNum;$m++)
    {
        // calculate weighting factor for each triangle

        $wPos[$m] = ($sliceFaceArea[$m] / $sliceFacetotArea);
        $sfcPosVec = $sliceFaceCenter[$m];


        // weight each triangle center by its area

        $sfcPosX[$m] = (($sfcPosVec.x) * $wPos[$m]);
        $sfcPosY[$m] = (($sfcPosVec.y) * $wPos[$m]);
        $sfcPosZ[$m] = (($sfcPosVec.z) * $wPos[$m]);


        // caluclate X, Y, Z coordinates of slice center

        $sfcPosSumX += $sfcPosX[$m];
        $sfcPosSumY += $sfcPosY[$m];
        $sfcPosSumZ += $sfcPosZ[$m];

        $wPosSum += $wPos[$m];
    }

    select -clear;
    select $selection;

    // update ProgressBar

    progressBar -edit
        -status "Estimating Line of Action"
        -step $i $gMainProgressBar;

    // set center position for slice $i

    $centerPos = <<($sfcPosSumX), ($sfcPosSumY), ($sfcPosSumZ)>>;
    $sliceCenterPos[$i] = $centerPos;


    // creates locator at the centroid of slice $i and groups all into the centroid locator group

    if ($createLOCs == 1)
    {
        $tempLoc = $nClean + "_Centroid_Slice" + $i;
        $centroidGRP = $nClean + "_LOC_GRP";
        spaceLocator - p 0 0 0 -n $tempLoc;
        select $tempLoc;
        move -r ($centerPos.x) ($centerPos.y) ($centerPos.z);
        select -clear;
        parent $tempLoc $centroidGRP;
    }
}

select -clear;
resetPolySelectConstraint;

progressBar -edit
    -endProgress
    $gMainProgressBar;


/**********  CREATES LOA BASED ON MUSCLE SLICES  ***********/


// create variables for LOA creation

string $n = $nClean;
```

```
string $nc = $n + "_path";
string $np = $n + "_poly";
string $npf = $np + "Shape.f[0]";
string $nGRP = $n + "_LOA_GRP";

string $CRVPtsArray[];
string $NumArray[];
string $t[];

int $j = 0;


// read slice center points into array for curve construction

clear $CRVPtsArray;

for ($j = 0;$j<$numberOfDivisions + 1;$j++)
{
    tokenizeList($sliceCenterPos[$j], $t);
    $CRVPtsArray[($j*4)] = "-p";
    $CRVPtsArray[($j*4)+1] = $t[0];
    $CRVPtsArray[($j*4)+2] = $t[1];
    $CRVPtsArray[($j*4)+3] = $t[2];
}

clear $NumArray;

for ($k = 0;$k<$numberOfDivisions - 1;$k++)
{
    $NumArray[($k*2)] = "-k";
    $NumArray[($k*2)+1] = $k;
}


// create strings for curve construction (i.e. curve points and knots)

string $curveDataAsString = stringArrayToString($CRVPtsArray," ");
string $NumArrayAsString = stringArrayToString($NumArray," ");
string $curveString = $curveDataAsString + " -k 0 -k 0 " + $NumArrayAsString + " -k " +
($numberOfDivisions - 2) + " -k " + ($numberOfDivisions - 2);


// creat curve based on slice center position

eval curve -d 3 $curveString -n $nc;
reverseCurve -rpo 1 $nc;
rebuildCurve -rpo 1 -rt 0 -end 1 -kr 0 -kcp 0 -kep 1 -kt 1 -s 0 -d 1 -tol 1e-08 $nc;
rebuildCurve -rpo 1 -rt 0 -end 1 -kr 0 -kcp 0 -kep 1 -kt 1 -s 0 -d 3 -tol 1e-08 $nc;


// creates temporary helper to aim polyDisc along curve

spaceLocator - p 0 0 0 -n helper;
select helper;
move -r $CRVPtsArray[($numberOfDivisions*4)-3] $CRVPtsArray[($numberOfDivisions*4)-2]
$CRVPtsArray[($numberOfDivisions*4)-1];
select -clear;


// creates polyDisc to extrude onto curve

polyDisc -sides 12 -subdivisionMode 4 -subdivisions 0 - radius $LOARadius;
rename "pDisc1" $np;
select $np;
move -r $CRVPtsArray[($numberOfDivisions*4)+1] $CRVPtsArray[($numberOfDivisions*4)+2]
$CRVPtsArray[($numberOfDivisions*4)+3];
select -clear;


// aim polyDisc along first curve point and extrude it onto curve

aimConstraint -offset 0 0 0 -weight 1 -aimVector 0 1 0 -upVector 1 0 0 -worldUpType "scene" -n
aimhelper helper $np;
polyExtrudeFacet - kft 1 -divisions ($numberOfDivisions * 2) -inputCurve $nc $npf;
```

```
// clean up

delete helper aimhelper $muscle;
rename $nCopy $nClean;
select $np $muscle;
makeIdentity -apply true -t 1 -r 1 -s 1 -n 0 -pn 1;
doBakeNonDefHistory( 1, {"prePost" });
group -n $nGRP $nc $np;

if ($createLOCs == 1)
{
    $centroidGRP = $nClean + "_LOC_GRP";
    parent $centroidGRP $nGRP;
}

select -clear;
select $selection;
```

Supplementary References

1. Cuff, A. R. *et al.* Relating neuromuscular control to functional anatomy of limb muscles in extant archosaurs. *J. Morphol.* **280**, 666–680 (2019).

2. Wiseman, A. L. A. *et al.* Musculoskeletal modelling of the Nile crocodile (*Crocodylus niloticus*) hindlimb: Effects of limb posture on leverage during terrestrial locomotion. *J. Anat.* joa.13431 (2021) doi:10.1111/joa.13431.

3. Gignac, P. M. *et al.* Diffusible iodine-based contrast-enhanced computed tomography (diceCT): an emerging tool for rapid, high-resolution, 3-D imaging of metazoan soft tissues. *J. Anat.* **228**, 889–909 (2016).

4. Demuth, O. E., Rayfield, E. J. & Hutchinson, J. R. 3D hindlimb joint mobility of the stem-archosaur *Euparkeria capensis* with implications for postural evolution within Archosauria. *Sci. Rep.* **10**, 15357 (2020).

5. van Beesel, J., Hutchinson, J. R., Hublin, J. & Melillo, S. M. Exploring the functional morphology of the *Gorilla* shoulder through musculoskeletal modelling. *J. Anat.* **239**, 207–227 (2021).

6. Catmull, E. & Clark, J. Recursively generated B-spline surfaces on arbitrary topological meshes. *Comput. Des.* **10**, 350–355 (1978).

7. Hutchinson, J. R. The evolution of pelvic osteology and soft tissues on the line to extant birds (Neornithes). *Zool. J. Linn. Soc.* **131**, 123–168 (2001).

8. Hutchinson, J. R. The evolution of femoral osteology and soft tissues on the line to extant birds (Neornithes). *Zool. J. Linn. Soc.* **131**, 169–197 (2001).

9. Carrano, M. T. & Hutchinson, J. R. Pelvic and hindlimb musculature of *Tyrannosaurus rex* (Dinosauria: Theropoda). *J. Morphol.* **253**, 207–228 (2002).

10. Hutchinson, J. R. The evolution of hindlimb tendons and muscles on the line to crown-group birds. *Comp. Biochem. Physiol. Part A Mol. Integr. Physiol.* **133**, 1051–1086 (2002).

11. Molnar, J. L., Diogo, R., Hutchinson, J. R. & Pierce, S. E. Reconstructing pectoral appendicular muscle anatomy in fossil fish and tetrapods over the fins-to-limbs transition. *Biol. Rev.* **93**, 1077–1107 (2018).

12. Bishop, P. J., Cuff, A. R. & Hutchinson, J. R. How to build a dinosaur: Musculoskeletal modeling and simulation of locomotor biomechanics in extinct animals. *Paleobiology* **47**, 1–38 (2021).

13. Romer, A. S. The locomotor apparatus of certain primitive and mammal-like reptiles. *Bull. Am. Museum Nat. Hist.* **46**, 517–606 (1922).

14. Molnar, J. L., Pierce, S. E., Clack, J. A. & Hutchinson, J. R. Idealized landmark-based geometric reconstructions of poorly preserved fossil material: A case study of an early tetrapod vertebra. *Palaeontol. Electron.* **15**, 2T (2012).

15. Bommes, D., Zimmer, H. & Kobbelt, L. Mixed-integer quadrangulation. *ACM Trans. Graph.* **28**, 1–10 (2009).

16. Bommes, D. *et al.* Quad-Mesh generation and processing: A Survey. *Comput. Graph. Forum* **32**, 51–76 (2013).

17. Rossoni, M., Barsanti, S., Colombo, G. & Guidi, G. Retopology and simplification of reality-based models for finite element analysis. *Comput. Aided. Des. Appl.* **17**, 525–546 (2020).
